# Serum EMMPRIN/CD147 promotes the lung pre-metastatic niche in a D2A1 mammary carcinoma mouse model

**DOI:** 10.3389/fimmu.2025.1568578

**Published:** 2025-04-30

**Authors:** Gabriele Feigelman, Elina Simanovich, Michal A. Rahat

**Affiliations:** ^1^ Immunotherapy Laboratory, Research Laboratories, Carmel Medical Center, Haifa, Israel; ^2^ Department of Immunology, Rappaport Faculty of Medicine, Technion-Israel Institute of Technology, Haifa, Israel

**Keywords:** EMMPRIN/CD147, pre-metastatic niche (PMN), fibroblasts, neutrophils, extracellular matrix remodeling, angiogenesis

## Abstract

Several types of cancer, including breast cancer, metastasize to the lung. However, before the disseminating tumor cells (DTCs) arrive there, the lung must be prepared as a hospitable environment for them, forming the pre-metastatic niche (PMN). It is now accepted that the primary tumor can release soluble mediators or extracellular vesicles that activate the PMN resident cells, recruit immune cells, promote angiogenesis, and remodel the extracellular matrix (ECM), even before the arrival of DTCs to the niche. However, not all the components of the tumor secretome are known. Here we demonstrate in a mouse model of breast cancer designed to generate lung PMN, that EMMPRIN, a multifunction protein and mediator of tumor-stroma cell interactions, is part of that secretome. To study the involvement of EMMPRIN in the generation of lung PMN, we knocked down its expression in D2A1 cells (D2A1-KD), treated the mice with the anti-EMMPRIN antibody developed in our lab (m161-pAb), or administered the recombinant EMMPRIN protein to healthy mice. We show that the primary tumor released elevated levels of EMMPRIN in mice implanted with paternal D2A1 cells (D2A1-WT), generating lung PMN by increasing VEGF, MMP-9 and TGFβ secretion, enhancing angiogenesis, activating fibroblasts, increasing neutrophils infiltration, and remodeling the ECM. These effects were inhibited in mice implanted with D2A1-KD cells or administered with m161-pAb. In healthy mice, the recombinant EMMRPIN recapitulated the effects of high EMMPRIN levels. Thus, EMMPRIN as part of the tumor secretome is sufficient to promote the lung PMN, and targeting it could potentially inhibit the metastatic cascade.

## 1 Introduction

Breast cancer (BC) remains the most prevalent malignancy in women worldwide and the leading cause of cancer-related deaths among females (1, 2). Although metastasis is a rare event, as only 0.01% of circulating tumor cells eventually form macro-metastases in tissues (3), the mortality rate, which was reduced due to early diagnosis, is still mostly due to metastasis (4). At primary diagnosis, up to 40% of the patients are found with disseminating tumor cells in their bone marrow, but only 6-10% of them present with metastatic disease (5). About one third of BC patients, especially those with tumors positive for estrogen receptor expression (ER^+^), eventually develop a metastatic disease in distant organs, years after the diagnosis of the primary tumor, suggesting that the metastatic cells remain dormant for many years despite adjuvant therapy (6). BC cells most commonly metastasize to the bones, lungs, liver, and brain (2), and about 15-20% of breast cancer patients eventually develop lung metastases, the second most common type of breast cancer metastasis after bone (7). Several effective treatment modalities that target proliferating cells, are routinely used for the primary tumors (e.g., tumor resection, chemotherapy, radiotherapy), but there are yet no effective treatments for metastasis. Therefore, there is an urgent need to target metastatic cells in remote organs, or disrupt their ability to colonize remote organs, to interfere with the metastatic cascade and prevent the formation of metastatic lesions.

The process of metastasis is complex and involves many stages. Some of the primary tumors cells can undergo epithelial-to-mesenchymal transition (EMT) and disseminate in very early stages of their development (8), but only a small proportion of them arrive and colonize the metastatic site to become disseminating tumor cells (DTCs) (9). These colonizing cells may lie dormant for months and years until they can overcome the suppressive signals in the remote organ, and resume their proliferation, finally yielding a metastatic lesion (8). However, the arrival and colonization of DTCs is not a random process, and it requires the preparation of the tissue to facilitate the homing and survival of the DTCs (2). Thus, according to Paget’s theory, the tissue’s normal microenvironment, or the “soil”, must change to become receptive to the DTC “seed”, in a process that generates the pre-metastatic niche (PMN) (10).

Presently, the changes made in the normal remote organ that turn it into a PMN are beginning to be elucidated. These include increased vascular leakage and angiogenesis that promote DTCs extravasation and colonization, recruitment of immune cells and activation of resident cells to inhibit anti-tumor immunity, and extracellular matrix (ECM) remodeling that changes the composition of the ECM proteins and increase tissue stiffness (11).

The process of PMN formation is triggered by the systemic secretion of soluble factors from the primary tumor (tumor secretome), including proteases, enzymes, cytokines, chemokines and metabolites. Other factors are released from the primary tumor in vesicles, particularly in exosomes, which contain microRNA, proteases, pro-angiogenic factors, and integrin molecules that help determine organotropism (11–13). Collectively, all these factors trigger the necessary processes that generate the PMN as a permissive microenvironment for DTCs colonization (2). However, the full spectrum of molecules that comprise the tumor secretome is not yet fully known.

Several cytokines and chemokines have already been implicated in the process of PMN formation. For example, lung endothelial cells, in response to VEGF secreted from experimental or human tumors, secrete enhanced levels of matrix metalloproteinase 9 (MMP-9), even before tumor cells spread, and render the lung tissue more receptive to tumor cell invasion (10). Additionally, tumor-secreted VEGF, transforming growth factor β (TGFβ), tumor necrosis factor α (TNFα) and CCL2 can induce lung angiogenesis and trigger local inflammation. These cytokines induce the expression of serum amyloid A (SAA), S100 calcium-binding proteins A8 and A9 (S100A8/S100A9), and MMPs in the lung tissue-resident cells (e.g., fibroblasts, lung epithelial cells). This, in turn, helps recruit immune cells, such as myeloid-derived suppression cells (MDSCs), neutrophils, tumor-associated macrophages (TAMs) and T regulatory cells (Tregs), which further secrete S100 proteins and MMPs, and increase vascular permeability in the lung (2, 11, 14, 15). Other factors that are secreted by the primary tumor, such as G-CSF, angiopoietin-like protein 2 (ANGPTL2), IL-6, and CCL2, promote the expansion of the myeloid lineage, their recruitment into the lung PMN and their polarization into an immunosuppressive phenotype (11). Mediators that are first secreted by the primary tumor and then by activated resident cells and recruited immune cells (e.g., prostaglandins, nitric oxide, MMPs, VEGF) enhance vascular permeability and angiogenesis, which is critical for the adhesion and extravasation of bone-marrow derived cells into the remote tissue, and create a pro-tumoral and immunosuppressive microenvironment (11). Activated resident fibroblasts can secrete CXCL12/SDF1 to recruit Tregs and promote the PMN immunosuppressive microenvironment (11). However, the comprehensive tumor secretome is far from being fully characterized.

Another characteristic of the PMN is extensive ECM remodeling. The composition of the ECM is specific to each organ, and is generated primarily by resident fibroblasts, endothelial cells, and infiltrating immune cells. In the PMN, these proteins are modified by the same cells once they are activated. Different types of collagens (Col), especially Col6A, fibronectin and laminin are among the top ECM proteins in healthy mouse lungs (16). Mediators secreted from the primary tumor can activate lung fibroblasts to deposit more fibronectin, periostin and lysyl oxidase (LOX), thus enhancing the attachment of bone marrow derived cells (BMDCs) and of DTCs (11, 14). The LOX family of proteins that is responsible for the crosslinking of collagen fibers, and the increased expression of the tissue inhibitor of metalloproteinases-1 (TIMP-1) that inhibits the activity of MMPs, contribute to the enhanced stiffness of the tissue. This, in turn, enhances fibroblast activation and promotes tumor cell signaling, survival and proliferation (11, 12). The stiff ECM is sensed by integrin β1 and α5, and induces the expression of LOX family proteins, that in turn activate lung fibroblasts to produce more fibronectin, MMP-9, and CXCL12/SDF1, and facilitate the recruitment of bone marrow derived cells (BMDCs) (17). On the other hand, MMPs secreted by immune cells and fibroblasts can degrade the ECM proteins to facilitate the colonization of DTCs and angiogenesis. Other ECM proteins, such as versican, periostin, tenascin C, collagens types 1, 3, 4 and 6, as well as the ECM degrading protease MMP-9, have already been associated with formation of the lung PMN (18).

Extracellular matrix metalloproteinase inducer (EMMPRIN, or CD147) is a heavily glycosylated transmembranal protein that mediates cell-matrix and cell-cell interactions between epithelial cells, fibroblasts and immune cells (19). It is expressed on the surface of many cell types, but its expression is markedly elevated in tumor cells of numerous types of cancer, especially on metastatic cells, correlating with poor prognosis (20–23). EMMPRIN in its membranal or secreted forms, was initially discovered as a pro-angiogenic factor, because of its homophilic interactions that can induce the expression of the potent pro-angiogenic factors MMPs and VEGF (24, 25). However, it is now clear that, depending on the proteins it interacts with, EMMPRIN has many additional functions that promote tumor cell proliferation, metabolic reprograming, cell survival, chemotaxis, drug resistance, migration, invasion, stemness, and the EMT process (19, 26). For example, EMMPRIN can bind to CD44 to promote invasiveness (27), to cyclophilins to promote leukocyte adhesion and proliferation (28), and it can chaperone the lactate transporters MCT1 and MCT4 or bind to GLUT1 to facilitate metabolic reprograming (29). Thus, EMMPRIN is a mediator of tumor-stroma cells interactions, and an orchestrator of the cellular hallmarks of cancer (26, 30). This places EMMPRIN as an attractive candidate to target in order to inhibit tumor growth and metastasis. In fact, metuximab and metuzumab are two anti-human EMMPRIN antibodies that are currently in use or in clinical trials for the treatment of hepatocellular carcinoma (HCC) (31) and non-small cell lung carcinoma (NSCLC) (32), respectively, indicating the feasibility of EMMPRIN as a target. However, to the best of our knowledge, EMMPRIN has not yet been shown to be relevant in the generation of the PMN.

We, therefore, hypothesized that tumor-derived soluble EMMPRIN can trigger the generation of the lung PMN by promoting angiogenesis, immune cell recruitment, fibroblast activation and changes to the ECM. To this end, we evaluated the effects of EMMPRIN on the changes in the expression and accumulation of selected molecules and cells that take part in the processes described above in a new mouse model of the lung PMN.

## 2 Materials and methods

### 2.1 Cell cultures

The murine D2A1 cell line, derived from the D2 hyperplastic alveolar nodule mammary tumor cells (33), already expressing mCherry as a reporter gene, were used (kindly provided by Dr. Dalit Barkan, University of Haifa, Haifa, Israel). These parental cells served as a basis for the knockdown EMMPRIN procedure as described below in the D2A1-KD cells. All cells were cultured in high glucose Dulbecco’s Modified Eagle’s Medium (DMEM), with 10% fetal calf serum (FCS), 1% penicillin/streptomycin, 1% amphotericin B (full medium, all reagents from IMBH-Import and marketing, Beit HaEmek, Israel). The mouse brain endothelial cell line bEND3 (ATCC CRL-2299) was cultured in the same full medium that contained 1% glutamine, whereas D2A1 full medium contained 2% glutamine. All cells were split every 3–4 days at a ratio of 1:4 using trypsin-EDTA, routinely checked for the presence of mycoplasma, and used at passages 3-15. Cells were incubated at 37 °C and 5% CO2.

### 2.2 Preparation of D2A1 knocked-down cells

We applied the previously described procedure to knock-down EMMPRIN expression in the D2A1 cell line (34). Briefly, the parental D2A1 (D2A1-WT) cell line was infected with a lentivirus vector containing four mouse EMMPRIN siRNAs (**Supplementary Table S1**, Applied Biological Materials, Richmond, BC, Canada) at a multiplicity of infection (MOI) 135 with polybrene (8 μg/ml, Merck, Rathway, NJ, USA). This generated the D2A1-KD cells. Following infection, cells were cultured for 3 weeks in full medium with the addition of puromycin (15 μg/ml), and the medium was changed every 3–4 days to select for positive cells. The vector did not contain the immunogenic GFP marker (35), and therefore, we used limiting dilutions to isolate sub-clones with similar EMMPRIN expression. EMMPRIN expression in these sub-clones was then validated at the mRNA and protein levels (**Supplementary Figure S1**).

### 2.3 Experimental mouse models

BALB/c mice (female, 8 weeks old, Harlan, Jerusalem, Israel) were housed under specific pathogen-free conditions in the Pre-Clinical Research Authority (PCRA) Facility of IIT, accredited by AAALAC International, and kept with a 12 h light/dark cycle and access to food and water *ad libitum*. All protocols in this study were approved by the Committee on the Ethics of Animal Experiments of The Technion - Israel Institute of Technology (IIT), Haifa, Israel, in compliance with Israeli law and the Guide for the Care and Use of Laboratory Animals published by the US National Institutes of Health (IACUC permit number – IL-1140723). All personnel handling animals were trained in accordance with IIT and AAALAC standards.

#### 2.3.1 D2A1 spontaneous metastatic model

To examine their metastatic potential, 2x10^5^ D2A1-WT cells were orthotopically injected into the fourth mammary fat pad of BALB/c female mice. After 17 days, when the primary tumor reached a volume of about 100 mm^3^, the tumors were surgically resected, and the mice were euthanized on day 54 after implantation. Lungs were collected and the number of tumor nodules on the lung surface was grossly counted. Metastases in lung sections were detected by immunohistochemistry of mCherry.

#### 2.3.2 D2A1 experimental metastasis model

To determine whether the generation of the lung PMN really translates into increased metastasis, we performed an experimental metastasis model, by injecting D2A1-WT cells (5x10^5^ cells) intravenously on day 16 into healthy mice or into mice that were first implanted with D2A1-WT cells (2x10^5^ cells) at day 0. Mice were euthanized on day 25, to allow metastases to establish. Lungs were collected and the number of tumor nodules and their area was determined by H&E staining.

#### 2.3.3 D2A1 mouse PMN model and experimental groups

To generate a model of pre-metastatic niche, D2A1-WT or D2A1-KD (2x10^5^ cells each) were orthotopically injected into the fourth mammary fat pad of BALB/c female mice, 7–8 weeks old (Harlan). To simulate the effect of soluble EMMPRIN on the lung niche, healthy mice (Healthy) were intraperitoneally (i.p.) injected with 400 ng/ml/100μL of mouse recombinant EMMPRIN (R&D systems, Minneapolis, MN, USA) once every four days, starting on day 4, for a total of 6 injections. To neutralize EMMPRIN in D2A1-WT tumor-bearing mice, the anti-EMMPRIN antibody (10 μg/ml/100μL m161-pAb, generated in our lab) was i.p. injected once a week, starting on day 7, for a total of 3 injections. On day 28 following tumor cells implantation, mice were sacrificed. Tumors, lungs and blood were collected for subsequent analyses. Tumor nodules formed on the lung surfaces were macroscopically determined and counted. Data represent 2–3 biological repetitions in each group.

### 2.4 Real-time PCR (qPCR)

Total RNA was extracted from the lungs with the Total RNA Purification Kit (Norgen Biotek Corp, Thorold, ON, Canada) according to the manufacturer’s instructions. RNA concentrations were determined using the NanoDrop-One 1000 spectrophotometer (Thermo Scientific, Waltham, MA, USA). The cDNA was synthesized from 4 μg of total RNA using the FIREScript RT cDNA synthesis Mix with oligo (dT) and random primers kit (Solis BioDyne, Tartu, Estonia) according to manufacturer’s instructions. Then, 70 ng of the cDNA were amplified in duplicates in the StepOne system (Applied Biosystems, Foster City, CA, USA), with the 5X HOT FIREPol EvaGreen qPCR Mix Plus (Solis BioDyne) and 200 nM of the primers (**Supplementary Table S2**). To measure the relative quantity (RQ) of mRNA expression, each gene was amplified with an initial activation at 95°C for 12 min, followed by 40 cycles of 95°C for 15 sec, 60°C for 20 sec, and 72°C for 20 sec. The RQ was calculated in comparison to the endogenous reference gene GAPDH by the comparative ^ΔΔ^CT method. To compare the RQ between samples, the lungs obtained from healthy mice were used as calibrators.

### 2.5 H&E staining and immunohistochemistry

Lungs and primary tumors were formalin fixed and paraffin embedded (FFPE), and then cut into 4 μm thick sections. The sections were deparrafinized with the xylene substitute K-clear plus (Kaltek, Saonara, Italy), and rehydrated with decreasing ethanol concentrations. For Hematoxylin and eosin (H&E) staining, sections were first stained with hematoxylin (Gil III, 1:10 diluted, Leica Biosystems, Deer Park, IL, USA), rinsed with water and then with 1% eosin (Kaltek) and rinsed again. Slides were dehydrated again with ethanol and K-clear plus, and mounted with a coverslip and sealed with Pertex mounting medium (Histolab Products AB, Askim Gothenburg, Sweden). For immunohistochemistry, antigen retrieval was carried out for 15 min by microwave heating in citrate buffer pH 6.0. Hydrogen peroxide (3%) for 10 min incubation was used to quench the activity of endogenous peroxidase. Slides were then blocked with 2.5% BSA (for αSMA, Ly6G,mCherry) or with 1% BSA with 0.2% gelatin and 1% Tween-20 in PBS (for CD31) for 1 hour and incubated at 4°C overnight with the primary antibodies diluted as indicated (listed in **Supplementary Table S3**). After three washes (0.05% Tween 20 in PBS), the HRP-polymer anti-rabbit/rat was added for 1 h at room temperature, following additional three washes. The slides were then incubated for 5 min with the DAB substrate kit (Scytek Laboratories, Logan, UT, USA), rinsed with water and this step was repeated once more. All sections were then counterstained with hematoxylin Gil III (Leica Biosystems) and imaged using the bright field trinocular microscope (Olympus BX-60, Tokyo, Japan) and acquired with the MS60 camera and the MShot Image Analysis System V1 (MSHOT, Guangzhou Micro-shot Technology Co., Guangzhou, China).

### 2.6 Immunofluorescence

D2A1-WT and D2A1-KD cells (3x10^4^ cells/300μl full medium) were incubated overnight on sterile cover slips, then the medium was changed to a serum starvation medium, and cells were incubated for 48 h and then fixed with 300μL of 4% formaldehyde for 10 min. After fixation, cells were washed with PBS and blocked in buffer (2% donkey normal serum or BSA, in PBS) for 1 hour at room temperature. The primary antibody goat anti mouse EMMPRIN (diluted 1:200, R&D systems, Minneapolis, MN) was added and incubated in 120μL of the blocking buffer overnight at 4°C. After additional three washes with PBS, the secondary antibody donkey anti-goat Alexa Fluor^®^ 568 (diluted 1:1000) was incubated for 1 h in the dark at room temperature. Cells were washed again once in PBS, and then once with PBS with 10 nM DAPI for 5 min. Coverslips were mounted with Fluoromount G on carrying glass, and sealed with a nail polish.

### 2.7 Sandwich ELISA

The concentrations of mouse EMMPRIN were determined in the serum samples (diluted 1:100), in lung and in tumor lysates (normalized to the total protein), using the matched antibody pair kit (Abcam, Cambridge, UK). Additionally, the serum concentrations of TGFβ (diluted 1:90) and MMP-9 (diluted 1:200) and the lung concentrations of MMP-9, VEGF, TGFβ, IL-6 and TNFα in lung lysates (normalized to the total protein), were determined using the DuoSet ELISA kits (R&D Systems, Minneapolis, MN). Phosphorylation of ERK1/2 and STAT3 were assessed in lung lysates using the Intracellular DuoSet ELISA kits (R&D systems) and normalized to the total protein. All the assays were carried out according to manufacturers’ instructions.

### 2.8 Western blot analysis

Equal amounts of lung lysates (30 μg/lane) that were extracted in RIPA buffer were loaded on a gradient 4-20% SDS-PAGE, and proteins were transferred onto a nitrocellulose membrane (Advansta, San Jose, CA, USA) after electrophoresis. The Block-Chemi (Advansta) reagent was used to block the membranes for 1 hour, and then primary antibodies (listed in **Supplementary Table S4**) were diluted in blocking buffer as indicated overnight at 4°C. Following three washes in TBST buffer (1xTris-buffered saline with 0.1% Tween 20), the HRP-conjugated secondary antibodies were added (**Supplementary Table S4**) for 1 hour, and three more washes were performed. The membranes were then incubated with the WesternBright ECL HRP substrate (Advansta). Protein bands were visualized using the Omega Lum G imaging system (Aplegen, Pleasanton, CA, USA).

### 2.9 Assessment of total collagen content

The FFPE 4μm thick lung sections were deparaffinized and rehydrated as described in the immunohistochemistry section. Sections were then stained with Masson’s Modified Trichrome (Scytek) according to the manufacturer’s instructions. Briefly, sections were preheated in Bouin’s fluid for 1 h at 60°C, cooled for 10 min to room temperature and then rinsed with water. Then sections were stained with Weigert’s Iron Hematoxylin for 2 min, followed by rinsing with water for 2 min and the Biebrich Scarlet/Acid Fuchsin Solution was applied on the slides for 10 min and rinsed with water. To displace the red dye from the collagen fibers, the Phosphomolybdic/Phosphotungstic Acid Solution was applied for 10 min, and then the Aniline Blue Solution was applied for 7 min, rinsed in water and 1% acetic acid for 30 sec. Slides were dehydrated twice in 96% alcohol and twice in 100% alcohol for 2 minutes, cleared with xylene. Coverslips were mounted using Pertex mounting medium (Histolab Products AB, Gothenburg, Sweden). Total collagen content within 4 different fields per lung was assessed. Images were acquired as described in the immunohistochemistry section.

### 2.10 *In vitro* wound assay

The mouse endothelial cell line bEND3 was plated in a 96-well plate (4x10^4^ cells/well/100 μl) in full medium and grown to confluency overnight. A scratch was made with a pipette tip, and detached cells were washed away. Then, 5 μg of lung lysates diluted in 100 μl serum starvation media were applied unto the scratched endothelial layer. Images of the scratch were taken by an inverted microscope at the time of lysate addition (time 0h) and after 18 h (time 18h). The distance between the two sides of the scratch at 18h was measured using the ImageJ 1.53e software and subtracted from the distance at 0h to determine the migration length.

### 2.11 Statistical analyses

All experiments were conducted in 2–3 biological repetitions. Results are presented as mean ± standard error of mean (SEM). Statistical analyses were carried out using the GraphPad Prism software version 10.4.1 (GraphPad Software, Inc., Boston, MA, USA). Statistical significance between three groups was determined using one-way ANOVA followed by Bonferroni’s *post-hoc* multiple comparison test, and between two groups using the unpaired two-tailed non-parametric Mann-Whitney *t* test. Statistical significance was achieved at P-values less than 0.05 (α<0.05).

## 3 Results

### 3.1 EMMPRIN protein expression is reduced in D2A1-KD cells

One approach we took to reduce the tumor-derived secretion of EMMPRIN was to knockdown its expression by using a lentivirus carrying siRNA sequences, as described in the methods. We show that the expression of the EMMPRIN mRNA in the D2A1-KD cells was not changed relative to the paternal D2A1-WT cells (**Supplementary Figure S1A**). However, cellular protein expression as determined by IF, ELISA and WB demonstrated a reduction of 50% in protein expression (**Supplementary Figures S1B**, **S1D, E**), whereas the levels of EMMPRIN that accumulated in the supernatants of the D2A1-KD cells were reduced about 70% (**Supplementary Figure S1C**). The lack of change in EMMPRIN mRNA in comparison to the reduced EMMPRIN protein expression in the knocked down cells was also observed in our previous studies that used the mouse colon carcinoma cell line CT26 (34, 36), and suggests a post-translational regulation of EMMPRIN expression.

### 3.2 Using D2A1 cells as a model of lung PMN

The PMN by definition is a state where the metastatic tissue is remotely influenced by tumor cells located in the primary site, while there are no tumor cells in the tissue itself (14). Therefore, we needed an orthotopic model, where although the primary tumor can release metastatic cells that colonize the lung, a common site of metastasis, the time frame of the experiment is such that DTCs are absent from it. Hence, we chose the D2A1 mouse mammary carcinoma cell line, which generates metastasis at a low frequency. D2A1 cells were shown to develop macro-metastases in only two of five immunocompromised BALB/c nude mice (~ 40%), 4 weeks after orthotopic implantation (33). When D2A1 cells were implanted orthotopically into immunocompetent BALB/c mice, the majority of mice showed no macro-metastases, and when the orthotopic primary tumor was surgically resected, only a limited metastatic disease was observed (37).

**Figure 1:**
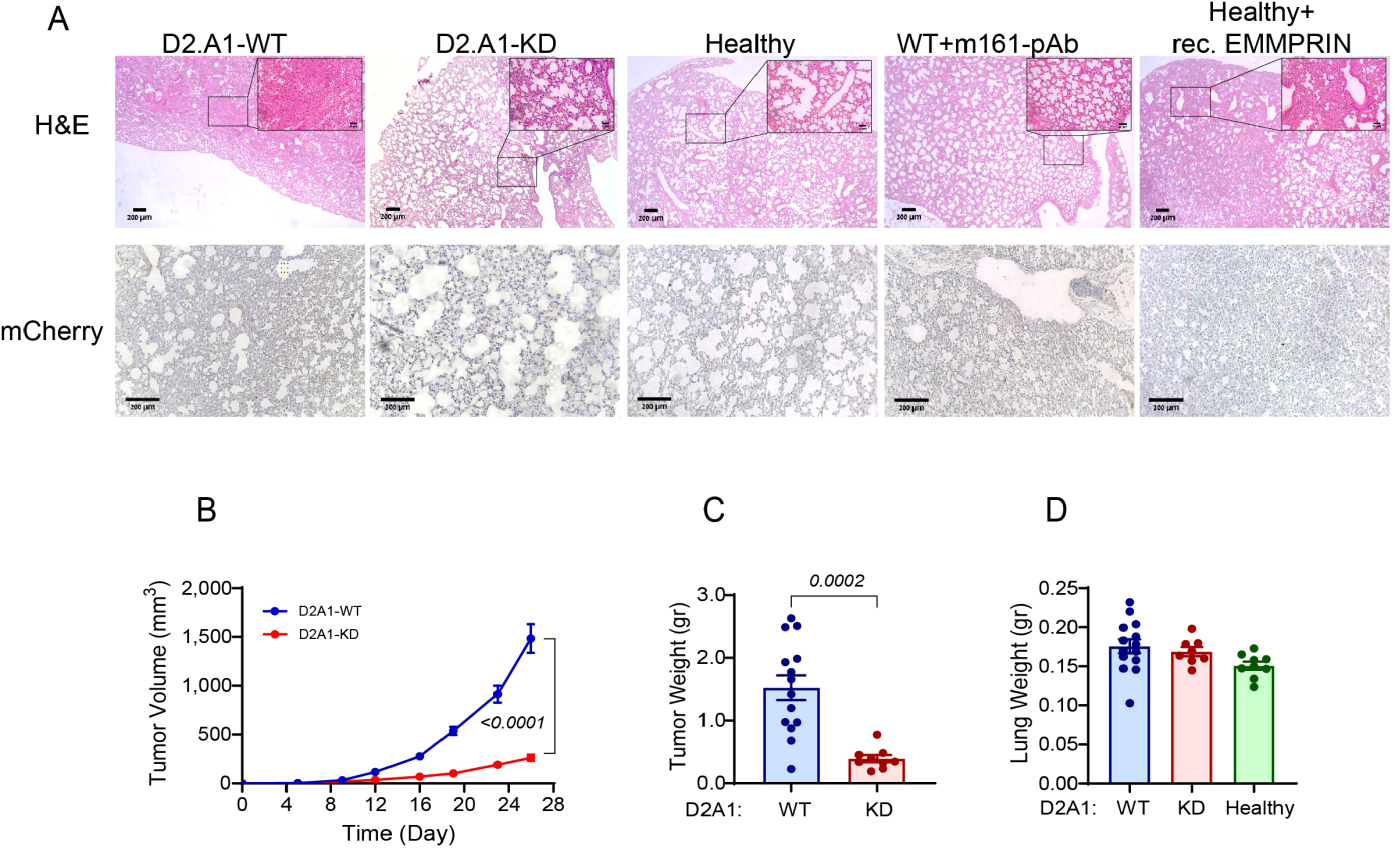
EMMPRIN promotes primary tumor growth, and D2A1 cells do not metastasize into the lung in this model. **(A)** Female BALB/c mice were orthotopically injected with D2A1-WT cells, with D2A1-KD cells (2x10^5^ cells each), or with no tumor cells (healthy). Alternatively, mice injected with D2A1-WT cells were i.p. treated with three injections of anti-EMMPRIN antibody (m161-pAb, 10 μg/ml/100μL, every 7 days), and healthy mice were i.p. injected with recombinant EMMPRIN (400 ng/ml/100μL, 6 times, every 4 days). After 28 days, mice were sacrificed, and their lungs were stained. **(A)** Representative images for the H&E staining (upper panel) or the immunohistochemical staining with the anti-mCherry antibody (lower panel). Bar size is 200 μm and 50 μm in the insets. The images demonstrate a denser and more congested lung structure in mice injected with the D2A1-WT cells or with the recombinant EMMPRIN, while mCherry remains unstained, indicating the lack of tumor cells in the lungs of all groups. **(B)** Tumor volumes or **(C)** tumor weights at the end of the experiment indicate that high levels of EMMPRIN promote tumor growth. **(D)** Wet lung weight was not changed, suggesting that metastatic mass was not added. Data are presented as mean ± SEM, and analyzed using one-way ANOVA followed by Bonferroni’s *post hoc* test, or the non-parametric two-tailed Mann-Whitney *t* test (n=14 for the D2A1-WT group, n=9 for the D2A1-KD group, n=9 for the healthy group, n=9 for the D2A1-WT + m161-pAb group, n=9 for the Healthy + rec. EMMRPIN group, in 2–3 biological repetitions).

Next, to demonstrate that the D2A1-WT cells we used maintain their metastatic potential, we orthotopically injected the D2A1-WT cells, and surgically excised the tumor at day 17. Fifty-four days after implantation, five out of ten immunocompetent BALB/c mice exhibited micro- or macro-metastases, as was shown by staining for mCherry (**Supplementary Figure S2C**). However, in mice that did not undergo surgery, the lungs remained free of metastases (**Supplementary Figure S2B**). As a positive control, we stained the primary tumors, which showed intense staining of the marker (**Supplementary Figure S2D**). These results verify that the D2A1-WT cells we used do have a metastatic potential upon resection, and that the lack of mCherry staining observed in the following experiments (**Figure 1A**, lower panel) truly indicates the absence of metastatic cells.

In numerous studies, the lung PMN is examined two weeks after implantation, when DTCs that colonized early did not have sufficient time to develop macro-metastasis. To ensure that our PMN model is genuine and that DTCs have sufficient time to develop into a visible metastatic lesion, we extended this period and orthotopically implanted the D2A1 cells or their derivatives into immunocompetent BALB/c mice without resecting the primary tumor, and allowed the experiment to last for 28 days before sacrificing the mice. As can be seen, no macro- or micro-metastases could be detected by either H&E staining or by staining for the reporter gene mCherry (**Figure 1A**, left panels). However, even without resection, the lung structure in mice implanted with D2A1-WT cells became denser and lost its typical alveoli structure that was easily observed in the healthy lung. Since the structural alterations were observed in the lungs in the absence of any detectable D2A1 metastatic cells, we concluded that this approach successfully modeled the lung PMN. Once the model for lung PMN was established, we continued in the following experiments to characterize the effects of EMMPRIN on the hallmarks of the lung PMN.

### 3.3 Elevated serum EMMPRIN levels promote tumor growth, change lung morphology, and correlate with tumor weight

We hypothesized that one of the mechanisms that allow the primary tumor to regulate the formation of the remote PMN is the secretion of EMMPRIN. To determine the involvement of soluble EMMPRIN in this process we took three alternative approaches. First, we knocked down EMMPRIN expression in the D2A1 cells (D2A1-KD) to reduce the circulating secreted levels of the protein. Secondly, we neutralized EMMPRIN by injecting the mice with the anti-EMMPRIN antibody (m161-pAb) that we have previously developed in our lab (38), and thirdly, we injected recombinant EMMPRIN protein into healthy mice to simulate the high serum levels of the protein. Using these three approaches, we evaluated the effects of the different EMMPRIN levels on the characteristics of the lung PMN.

First, we observed that the rate of the growth of the primary tumor and its final weight in mice injected with D2A1-KD cells were significantly reduced compared with mice injected with the D2A1-WT cells (**Figures 1B, C**). However, comparison of the wet lungs weight revealed no change between D2A1-WT cells, D2A1-KD cells and healthy mice that received no tumor cell implantation (**Figure 1D**). As metastases could change the wet weight of lungs, this was another indication for the absence of tumor cells and metastases in our model animals. Similar results were obtained when healthy mice were administered with the recombinant EMMPRIN or when mice implanted with D2A1-WT cells received the antibody treatment (data not shown).

The involvement of EMMPRIN in lung morphology was evident by the H&E staining (**Figure 1A**). Lungs of mice injected with the D2A1-KD cells, or lungs of mice injected with the D2A1-WT cells that received weekly injections of the m161-pAb were similar to the lungs of healthy mice, exhibiting classical structure of spacious alveoli. In contrast, lungs of mice that were injected with D2A1-WT cells, or lungs of healthy mice that were injected with recombinant EMMPRIN were denser and more congested, with fewer alveoli.

**Figure 2:**
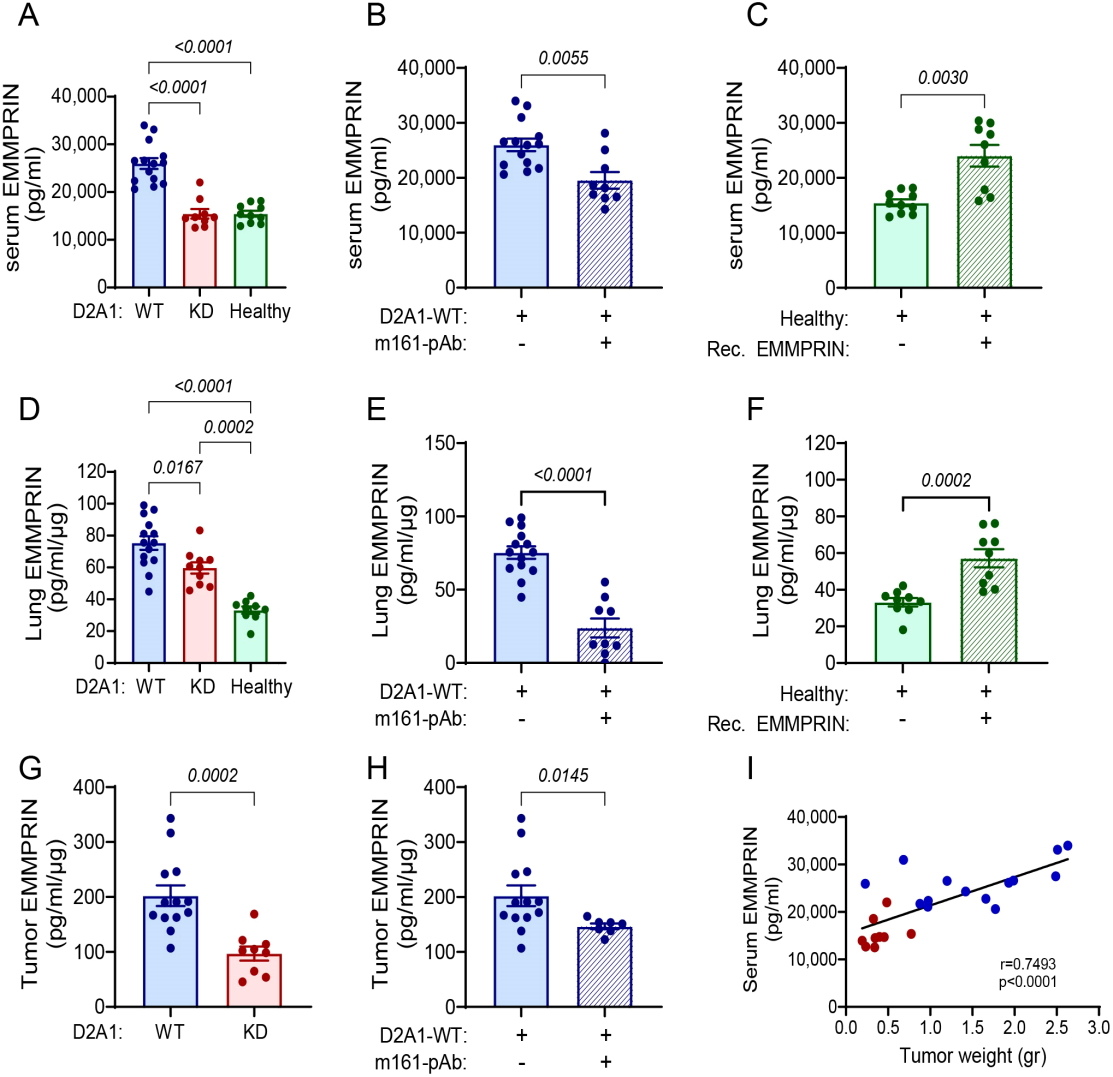
EMMPRIN serum levels correlate with tumor weight. Female BALB/c mice were orthotopically injected with D2A1-WT cells (n=13-14), with D2A1-KD cells (n=8-9), or with no tumor cells (healthy mice, n=9-10), or treated with the anti-EMMPRIN antibody (m161-pAb, n=9) or recombinant EMMPRIN protein (n=9) as described in the legend of **Figure 1**. Mice were sacrificed at day 28 and EMMPRIN levels were determined by ELISA in the **(A-C)** serum, **(D-F)** lung lysates, and **(G, H)** tumor lysates. Serum, lung and tumor EMMPRIN levels were increased in the group injected with D2A1-WT cells relative to the group injected with D2A1-KD cells or to healthy mice, and these levels could be inhibited using the anti-EMMPRIN antibody or recapitulated by injecting recombinant EMMPRIN. Data are presented as mean ± SEM. Significance between three groups is analyzed using one-way ANOVA followed by Bonferroni’s *post-hoc* test, and two groups are analyzed using the non-parametric two-tailed Mann-Whitney *t* test. **(I)** Serum EMMPRIN levels were correlated with the primary tumor weight using the non-parametric Spearman correlation test. Blue and red dots indicate mice implanted with D2A1-WT or D2A1-KD cells, respectively.

To examine our hypothesis that the primary tumor secretes EMMPRIN, which might affect the PMN, we determined the levels of EMMPRIN in the serum, in the lungs, and in the primary tumor. Serum levels of EMMPRIN in mice injected with D2A1-WT cells were the highest, and were markedly reduced by 40% in mice injected with the D2A1-KD cells, which were equivalent to those of the basal levels detected in healthy mice (**Figure 2A**). The administration of the neutralizing m161-pAb reduced serum EMMPRIN levels by 24% relative to the D2A1-WT mice (**Figure 2B**), and injections of recombinant EMMPRIN increased them by 55% relative to healthy mice and brought them to levels almost equivalent to those of the D2A1-WT mice (**Figure 2C**). Thus, the effects on serum EMMPRIN could be manipulated by using the antibody or the recombinant protein. Similar results were observed locally in the lung, where the mice injected with D2A1-KD cells exhibited reduced levels of lung EMMPRIN relative to mice injected with the D2A1-WT cells, the antibody reduced them and the recombinant EMMPRIN increased them (**Figures 2D-F**). Of note, the EMMPRIN levels in the lung lysates could reflect both the soluble EMMPRIN arriving to the lung in the serum, and the EMMPRIN produced by the resident lung cells. To distinguish between these two possible sources, we additionally stained the lungs for EMMPRIN by immunohistochemistry, examining only the expression in resident lung cells. We show that EMMPRIN expression levels remained unchanged between the lungs of healthy mice, mice implanted with D2A1-WT or mice implanted with D2A1-KD cells. Only in lungs carrying metastases, EMMPRIN expression levels in the tumor cells were increased (**Supplementary Figure S7**). Levels of EMMPRIN in lysates of the primary tumors of mice injected with the D2A1-KD cells were reduced by 50% compared to the levels in mice injected with the D2A1-WT cells, and the mice treated with the antibody showed 30% reduction compared to the D2A1-WT mice (**Figures 2G, H**). Most importantly, the serum levels of EMMPRIN in WT and KD mice positively correlated with the tumor weight (**Figure 2I**), suggesting that the source of the increased serum EMMPRIN, beyond the basal levels observed in healthy mice, was the primary tumor.

### 3.4 EMMRPIN enhances inflammatory cytokines in the lung PMN

To learn about the lung inflammatory microenvironment and the effects of EMMPRIN on it, we evaluated the levels of selected cytokines (VEGF, MMP-9, TGFβ) that are known to be important in the generation of the lung PMN (2, 11). First, we evaluated the serum cytokine levels. We did not assess the serum levels of VEGF, as these were mostly below the level of detection of our ELISA assay. The serum levels of MMP-9 were increased in mice implanted with the D2A1-WT cells relative to those implanted with the D2A1-KD cells or the healthy mice (**Supplementary Figure S3A**). Mice bearing D2A1-WT cells that were administered with the m161-pAb reduced their MMP-9 serum levels, while healthy mice injected with recombinant EMMPRIN increased their serum MMP-9 levels (**Supplementary Figures S3B, S3C**). In contrast, knocking-down EMMPRIN expression or administrating the recombinant EMMPRIN protein did not change the serum levels of TGFβ, whereas the anti-EMMPRIN antibody reduced them relative to the mice implanted with D2A1-WT cells (**Supplementary Figures S3D-S3F**).

We also determined the levels of these cytokines in the primary tumor lysates, to explore whether EMMPRIN affects the tumor secretome by influencing the production of those cytokines within the primary tumor. MMP-9 levels in the primary tumors of D2A1-WT implanted cells (63.5 ± 4.3 pg/ml/μg) were higher than those in the D2A1-KD implanted mice (41.2 ± 9.6 pg/ml/μg, p<0.0438), and the administration of the m161-pAb in D2A1-WT implanted mice produced even lower results (19.8 ± 4.1 pg/ml/μg. p<0.001 relative to the D2A1-WT tumors). In contrast, the TGFβ levels in the primary tumors of the D2A1-WT implanted cells (4.4 ± 0.63 pg/ml/μg) were the same as those in the D2A1-KD implanted mice (4.1 ± 0.45 pg/ml/μg), and the primary tumors in mice administered with the antibody (5.3 ± 0.9 pg/ml/μg). Similarly, VEGF levels in the primary tumors of D2A1-KD implanted mice were unchanged relative to the D2A1-KD implanted mice (2.6 ± 0.18 vs. 2.9 ± 0.8 pg/ml/μg), but the administration of the anti-EMMPRIN antibody reduced it by 2-fold (p<0.001).

**Figure 3:**
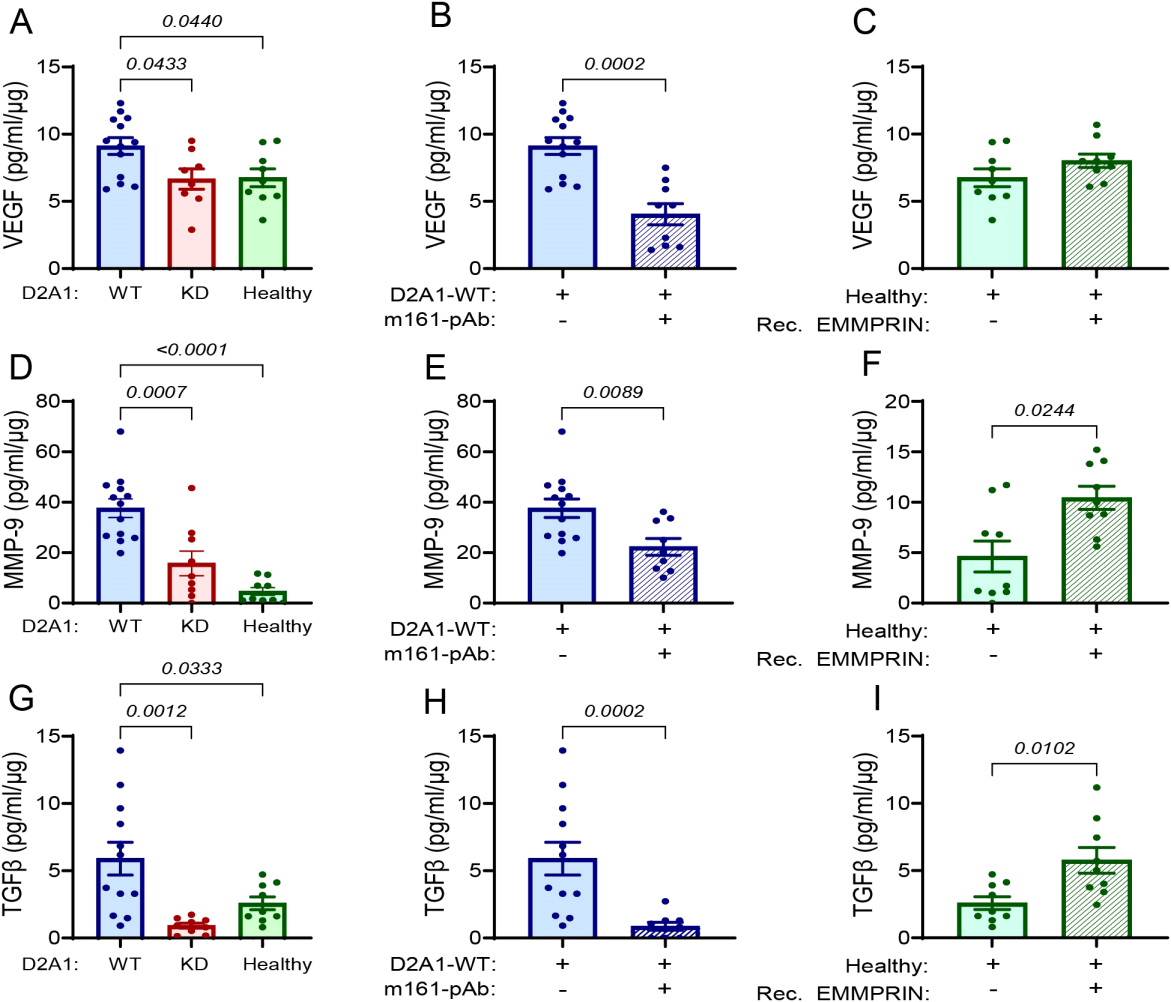
EMMPRIN promotes the secretion of VEGF, MMP-9 and TGFβ in the lung PMN. Mice were injected with the different tumor cells and treatments as described in the legend of **Figure 1**. The levels of **(A-C)** VEGF, **(D-F)** MMP-9, and **(G-I)** TGFβ, were evaluated in the lung lysates and normalized to the total protein (n=12–13 for the D2A1-WT group, n=8–9 for the D2A1-KD group, n=9 for the healthy group, n=9 for the D2A1-WT + m161-pAb group, n=9 for the Healthy + rec. EMMRPIN group). Data are presented as mean ± SEM. Three groups were analyzed using one-way ANOVA followed by Bonferroni’s *post-hoc* test, and two groups were compared using the non-parametric two-tailed Mann-Whitney *t* test. The three cytokines were increased in the group injected with D2A1-WT cells relative to the group injected with D2A1-KD cells or to healthy mice, and the involvement of EMMPRIN in their regulation was demonstrated by the use of the anti-EMMPRIN antibody or by injecting recombinant EMMPRIN.

In the lung, the effects were more noticeable. Relative to their lung levels in healthy mice, the levels in mice injected with the D2A1-WT cells were increased by 37% for VEGF (**Figure 3A**), by 8-folds for MMP-9 (**Figure 3D**), and by 2-folds for TGFβ (**Figure 3G**). The levels of the three cytokines in the lungs of mice injected with the D2A1-KD cells were no different from their levels in healthy mice. The administration of the m161-pAb reduced the levels of the three cytokines in mice injected with the D2A1-WT cells (**Figures 3B, E, H**), and the recombinant EMMPRIN protein increased them, except in the case of VEGF (**Figures 3C, F, I**).

To show that the effect was specific, we determined the level of pro-inflammatory cytokines that are not regulated by EMMPRIN. Relative to healthy mice, the implantation of the D2A1-WT cells increased the levels of IL-6, but not of TNFα, and the manipulation of EMMPRIN levels by the D2A1-KD cells, the m161-pAb or the recombinant protein had no effect on IL-6 or TNFα levels (**Supplementary Figures S3G-S3L**). Thus, EMMPRIN selectively affects cytokines in the lung PMN.

### 3.5 Elevated EMMPRIN levels promote angiogenesis within the lung PMN

**Figure 4:**
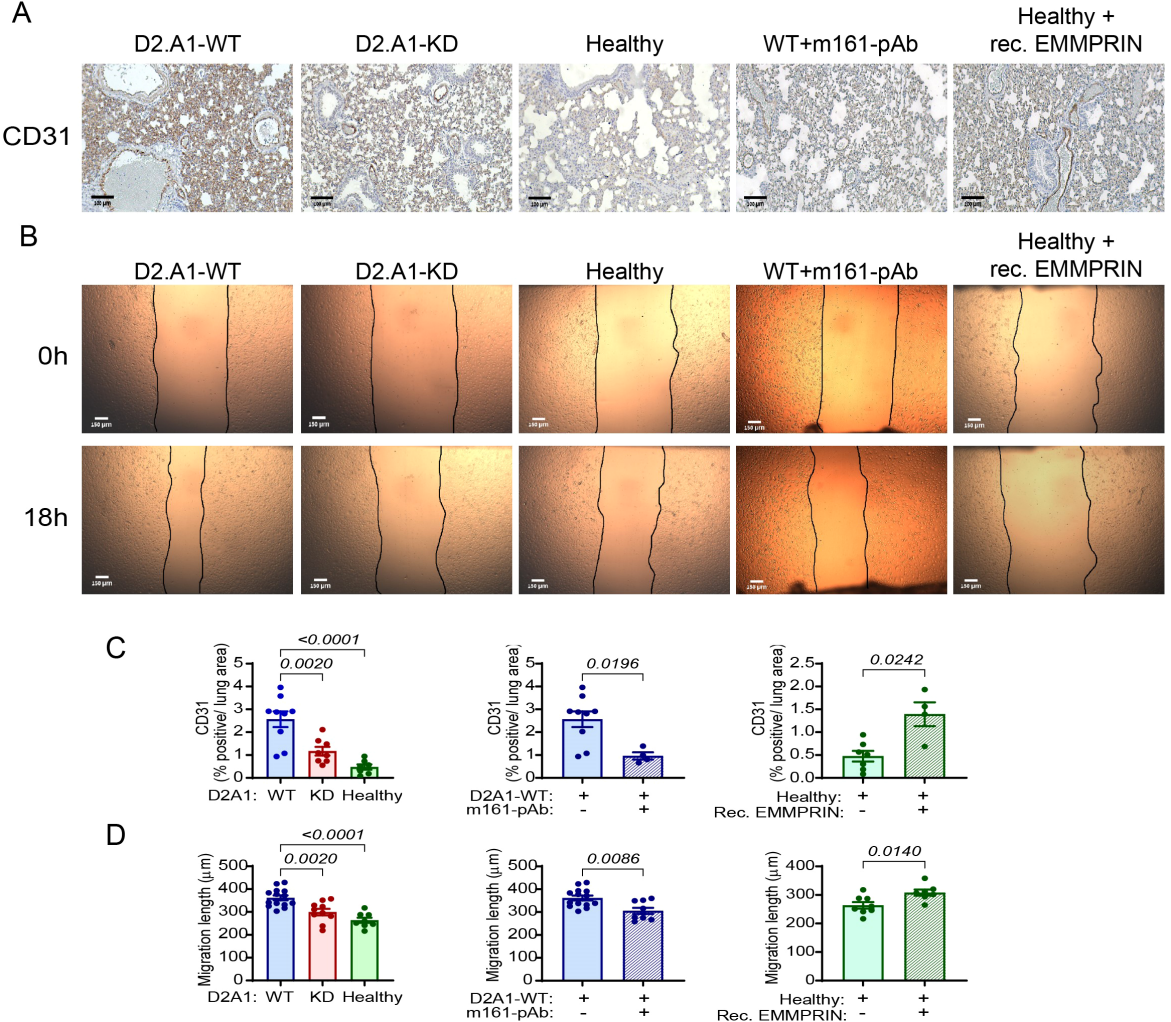
EMMPRIN promotes angiogenesis in the lung PMN. Mice were injected with the different tumor cells and treatments as described in the legend of **Figure 1**. **(A)** Representative images of the endothelial cell marker CD31 staining in the lungs, and **(C)** their quantitation (n=9 for the D2A1-WT group, n=8 for the D2A1-KD group, n=7 for the healthy group, n=7 for the D2A1-WT + m161-pAb group, n=9 for the Healthy + rec. EMMRPIN group). Bar size is 100 μm. **(B)** Representative images of bEND3 endothelial cell migration length after their incubation for 18 h with serum-starvation media containing 5µg of lung lysates from the different experimental groups. **(D)** Quantitation of the migration length (n=14 for the D2A1-WT group, n=10 for the D2A1-KD group, n=8 for the healthy group, n=9 for the D2A1-WT + m161-pAb group, n=7 for the Healthy + rec. EMMRPIN group). Bar size is 150 μm. Data are presented as mean ± SEM. Three groups were analyzed using one-way ANOVA followed by Bonferroni’s *post-hoc* test, and two groups were compared using the non-parametric two-tailed Mann-Whitney *t* test. The increased CD31 staining of endothelial cells in the mice implanted with the D2A1-WT cells and their longer migration distance suggests that they proliferate and migrate, two properties necessary for angiogenesis, more than the endothelial cells in healthy mice or mice implanted with the D2A1-KD cells. The importance of EMMPRIN to this process is exemplified by the results from the addition of h161-pAb or the injection of recombinant EMMPRIN.

Angiogenesis, the process of sprouting of new blood vessels from existing ones, is one of the hallmarks of the process of PMN generation, improving the infiltration of immune cells, mediators, and later the DTCs themselves, into the normal tissue. Angiogenesis requires the proliferation of endothelial cells and their arrangement into functional, although leaky vessels (39). Endothelial cells respond to stimulators such as VEGF and TGFβ, which trigger their proliferation and migration. To assess changes in angiogenesis in the lung PMN, we first stained lung sections from the different experimental groups with CD31, a marker of endothelial cells, and quantified the percentage of positively stained area from the total lung area. Relative to the small percentage of CD31^+^ cells in the lungs of healthy mice, mice injected with D2A1-WT cells demonstrated a 5-fold increase in endothelial cells, whereas no significant change was seen in endothelial cells in mice injected with the D2A1-KD (**Figures 4A, C**). The administration of the m161-pAb reduced the percentage of endothelial cells in the lungs of mice injected with the D2A1-WT cells, and the recombinant EMMPRIN protein enhanced their presence in healthy mice. Thus, the increased CD31 staining observed implies increase in the number of endothelial cells and enhanced lung angiogenesis, induced by EMMPRIN.

We next evaluated the angiogenic potential of the lung lysates, by applying them onto a scratched monolayer of mouse bEND3 endothelial cells and observing the distance the cells migrated to in order to close the gap. Endothelial cells that were treated with lung lysates derived from the D2A1-WT-injected mice that had elevated levels of EMMPRIN, VEGF, TGFβ and MMP-9, migrated to a longer distance (about 40%) than the bEND3 cells that were treated with lung lysates from healthy mice or from mice injected with the D2A1-KD cells (**Figures 4B, D**). The anti-EMMPRIN antibody decreased the angiogenic potential of lung lysates obtained from mice injected with the D2A1-WT cells, and the administration of the recombinant EMMPRIN protein increased it in healthy mice. These results re-confirm the effect of EMMPRIN on endothelial cells, and suggest that high levels of soluble EMMPRIN in the serum increase angiogenesis in the lung PMN.

### 3.6 Serum EMMPRIN changes the immune infiltrate and activates fibroblasts in the pre-metastatic lung

**Figure 5:**
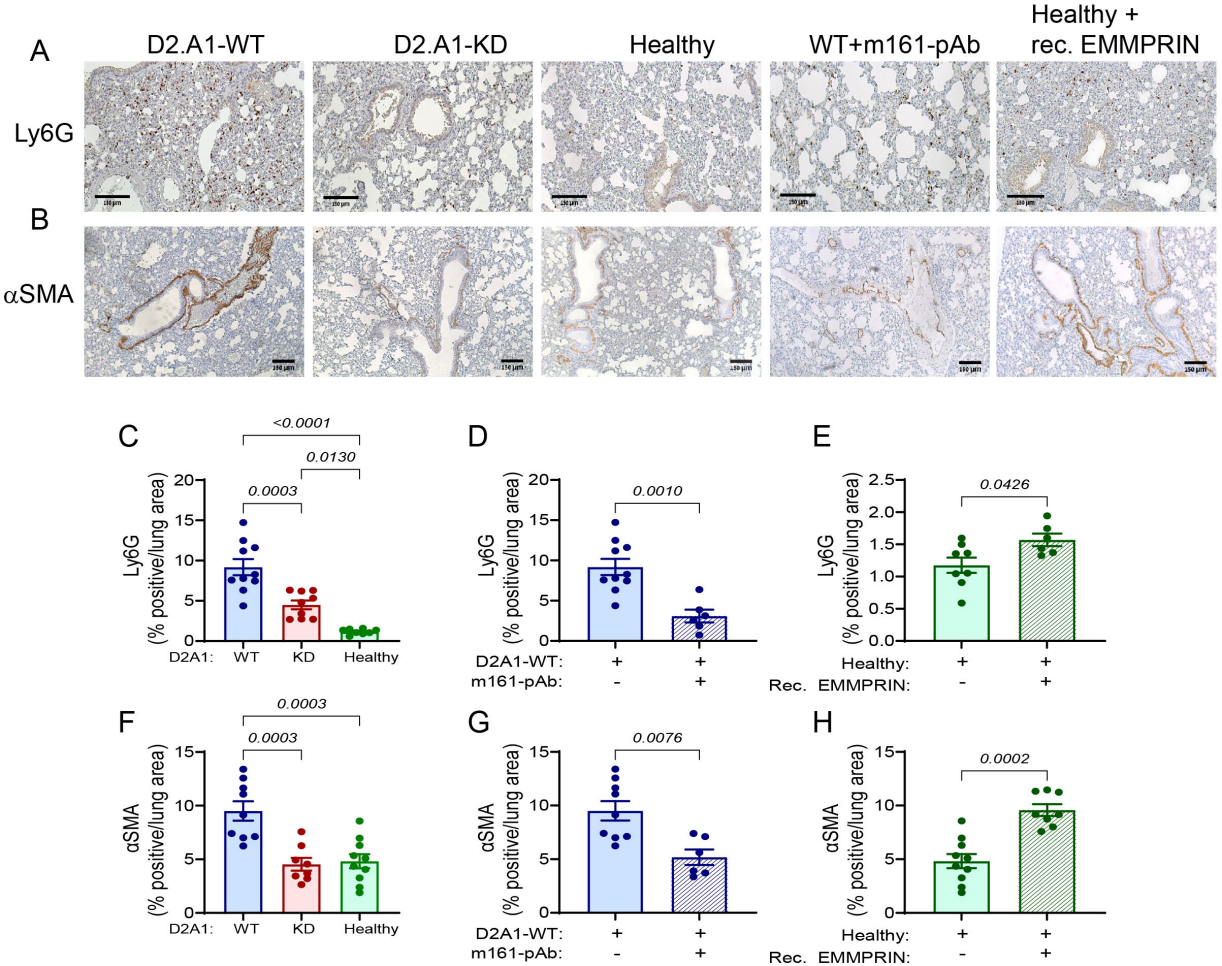
EMMPRIN promotes neutrophil infiltration and fibroblast activation in the lung PMN. Mice were injected with the different tumor cells and treatments as described in the legend of **Figure 1**. **(A)** Representative images of the neutrophil marker Ly6G and **(B)** of the fibroblasts activation marker αSMA staining in the lungs. Bar size is 150 μM. **(C-E)** Quantification of neutrophil infiltration, and **(F-H)** of fibroblast activation in the different experimental groups (n=9–10 for the D2A1-WT group, n=8–9 for the D2A1-KD group, n=8–10 for the healthy group, n=6 for the D2A1-WT + m161-pAb group, n=6–8 for the Healthy + rec. EMMRPIN group). Data are presented as mean ± SEM. Three groups were analyzed using one-way ANOVA followed by Bonferroni’s *post-hoc* test, and two groups were compared using the non-parametric two-tailed Mann-Whitney *t* test. Infiltrated neutrophils and fibroblasts were activated more in the lungs of mice implanted with the D2A1-WT cells than healthy mice or mice implanted with D2A1-KD cells. EMMPRIN’s involvement in these processes is also demonstrated by the results of the administration of the anti-EMMPRIN antibody and the injection of recombinant EMMPRIN.

The PMN is characterized by increased infiltration of immune cells. We therefore chose to examine two important stromal cell populations: neutrophils and fibroblasts. To assess whether circulating EMMPRIN affects neutrophil infiltration, we stained lung sections for the neutrophil marker Ly6G. Relative to the healthy mice, the lungs of mice implanted with D2A1-WT cells had an elevated number of neutrophils. Lungs from mice injected with the D2A1-KD cells had a reduced level of neutrophils compared to the D2A1-WT mice, but still higher than the neutrophils in the healthy mice (**Figures 5A, C**). The ability of the anti-EMMPRIN antibody to reduce the number of neutrophils relative to their D2A1-WT implanted control mice (**Figure 5D**), and the ability of the recombinant protein to increase this number relative to their healthy control mice (**Figure 5E**) further support the role of EMMPRIN in recruiting neutrophils to the PMN.

We next evaluated the ability of EMMPRIN to activate fibroblasts. First, we show that at the level of mRNA expression, the activated fibroblast marker PDGFRα is enhanced in the lungs of mice implanted with D2A1-WT cells relative to both healthy mice and D2A1-KD implanted mice, and that the m161-pAb reduced this expression, whereas the recombinant protein increased it (**Supplementary Figures S4D-S4F**). The same pattern was observed when we looked at another marker of activated fibroblasts, α-smooth muscle actin (αSMA, or by its gene name *ACTA2*) (**Supplementary Figures S4A-S4C**). To learn about the spatial localization of the cells, we stained the lung sections with the anti-αSMA antibody, and observed that most of the staining in all experimental groups was localized to the peribronchiole area (**Figure 5B**). A basal level of staining was observed in healthy mice and in mice implanted with D2A1-KD cells. However, mice injected with the D2A1-WT cells exhibited elevated levels of staining (**Figure 5F**), which were reduced back to the basal levels upon treatment with the anti-EMMPRIN antibody (**Figure 5G**). Conversely, injections of recombinant EMMPRIN elevated the levels of αSMA staining to those of the D2A1-WT implanted mice (**Figure 5H**). Thus, soluble EMMPRIN enhances neutrophil infiltration into the lungs and the activation of fibroblasts in the lungs, both typical to the generation of the PMN.

### 3.7 EMMPRIN changes the ECM composition in the pre-metastatic lung

**Figure 6:**
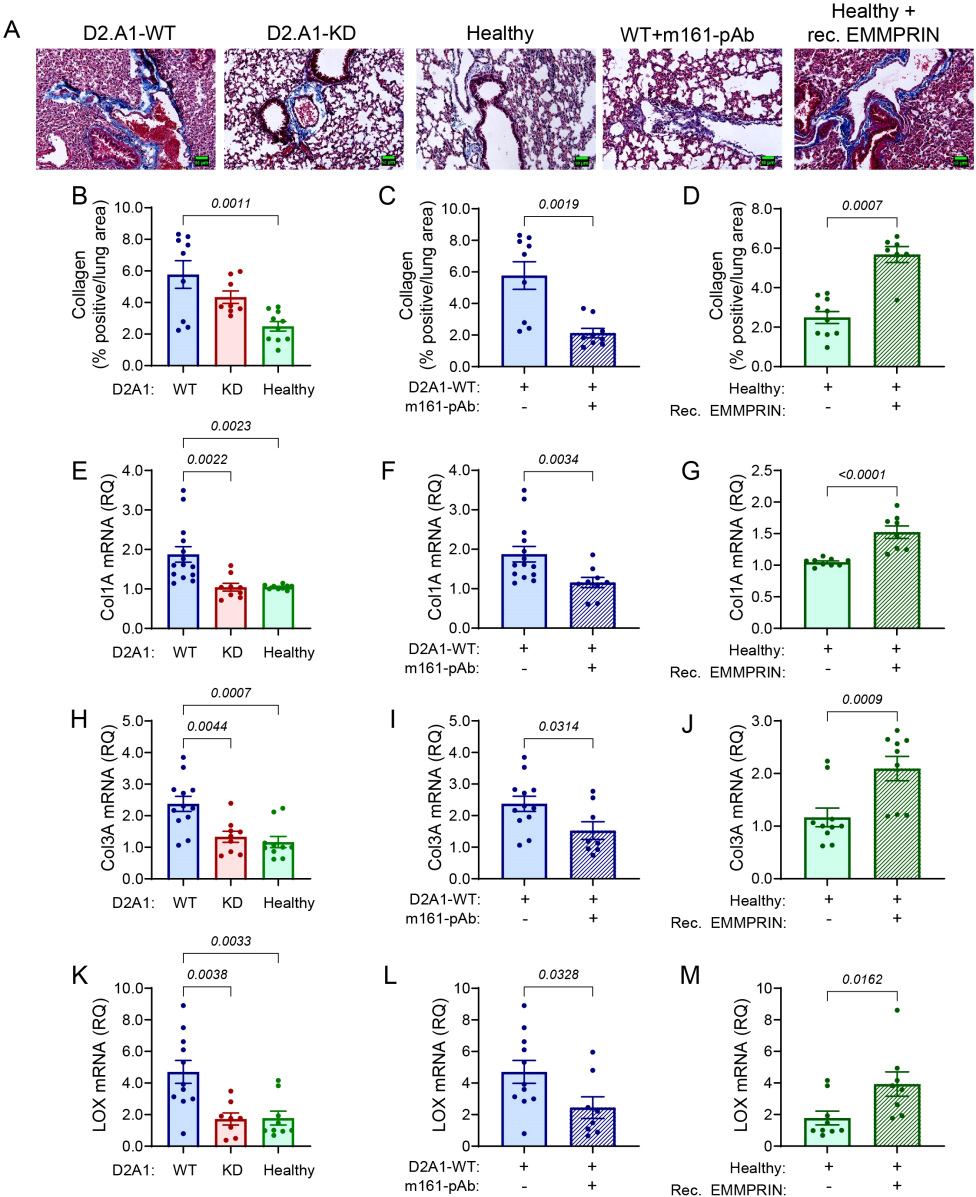
EMMPRIN regulates the expression of interstitial collagens. Mice were injected with the different tumor cells and treatments as described in the legend of **Figure 1**. **(A)** Representative images of lung sections from the different experimental groups that were stained with Masson’s Trichrome. Blue fibers indicate presence of collagen. Bar size is 150 μm. **(B-D)** Quantification of the collagen fibers stained by Masson’s Trichrome (n=9 for the D2A1-WT group, n=8 for the D2A1-KD group, n=9 for the healthy group, n=9 for the D2A1-WT + m161-pAb group, n=7 for the Healthy + rec. EMMRPIN group). Total RNA was extracted from the lungs, reverse transcribed and amplified for the determination of mRNA expression of **(E-G)** collagen 1A, **(H-J)** collagen 3A **(K-M)** LOX (n=11–12 for the D2A1-WT group, n=8–9 for the D2A1-KD group, n=9–10 for the healthy group, n=8–9 for the D2A1-WT + m161-pAb group, n=8–9 for the Healthy + rec. EMMRPIN group). Data are presented as mean ± SEM. Three groups were analyzed using one-way ANOVA followed by Bonferroni’s *post-hoc* test, and two groups were compared using the non-parametric two-tailed Mann-Whitney *t* test. The ECM in mice implanted with the D2A1-WT cells was denser with more collagen fibers, and the expression of interstitial collagens Col1A and Col3A, as well as the crosslinking enzyme LOX were higher than the healthy mice or mice implanted with D2A1-KD cells. Again, EMMPRIN’s involvement was demonstrated by the results of administration of the m161-pAb and the injection of recombinant EMMPRIN.

We next examined the effect of EMMPRIN on the expression of some lung ECM proteins. First, by staining the lung tissue with the Masson’s trichrome reagent, we demonstrate that relative to healthy mice, lung tissue becomes enriched with collagen fibers in mice implanted with the D2A1-WT cells and in mice injected with recombinant EMMPRIN. Mice implanted with the D2A1-KD cells exhibited reduced staining of collagen fibers, and mice implanted with the D2A1-WT cells that were injected with the m161-pAb antibody showed levels similar to healthy mice (**Figures 6A-D**). This pattern was also observed for the interstitial collagens (collagen 1A and collagen 3A) at the mRNA expression levels (**Figures 6E-J**). Moreover, the levels of the lysyl oxidase (LOX) mRNA, an enzyme which crosslinks collagen fibers and renders the tissue stiffer, were also regulated by EMMPRIN in the same manner (**Figures 6K-M**). Thus, we infer that EMMPRIN regulates interstitial collagens and increases lung tissue stiffness.

In contrast to these results, the accumulation of mRNAs coding for proteins that typically constitute the basement membrane (Col6A, Col4A, laminin subunit γ1), was unchanged by the manipulation of EMMPRIN levels using D2A1-KD cells, anti-EMMPRIN antibody or recombinant EMMPRIN (**Supplementary Figures S5E-S5M**). Of note, the accumulation of Col6A and laminin γ1 mRNAs were increased in the tumors generated by both D2A1-WT and D2A1-KD cells (**Supplementary Figures S5E, S5K**), suggesting that the presence of tumor cells increased the transcription of these genes in the lung in a non-EMMPRIN-dependent manner. However, when we examined the protein expression of Col6A, one of the abundant lung ECM proteins (16), we observed its reduction at the protein level in mice implanted with the D2A1-WT cells or in healthy mice that were injected with the recombinant EMMPRIN protein, relative to lungs of healthy mice. Conversely, mice implanted with D2A1-WT cells that were injected with the m161-pAb or mice implanted with the D2A1-KD cells had Col6A protein levels comparable to those of healthy mice (**Supplementary Figures 5SA-5SD**). Thus, although EMMPRIN does not regulate Col6A at the transcriptional level, it has a down-regulating effect at the post-transcriptional level.

### 3.8 EMMPRIN-induced lung PMN promotes the metastasis

Our previous results indicate that the presence of an orthotopic D2A1 tumor generated high serum EMMPRIN levels (**Figure 2A**) and a PMN in the lungs, as assessed by the increased secretion of lung cytokines, enhanced angiogenesis, increased neutrophil infiltration and fibroblasts activation, and ECM remodeling. However, despite the changes in the lung, D2A1 cells did not metastasize into the lung, raising the question of whether these changes could in fact functionally enhance metastasis. To answer this question, we used an experimental model of metastasis and injected the cells intravenously (i.v.), thereby circumventing the early stages of the metastatic cascade and directly modeling the final stages of colonization and proliferation of the tumor cells in the metastatic organ. D2A1-WT cells were orthotopically implanted in the mammary fat pad, and after 16 days these mice were i.v. injected with the same D2A1-WT cells. As a control group, we injected the D2A1-WT cells into healthy mice that did not bear any tumors. Mice from the two groups were sacrificed 25 days after orthotopic implantation, to allow the establishment of metastases. **Supplementary Figure S6** demonstrates that in healthy mice that did not have the chance to generate a PMN and were i.v. injected with the tumor cells, the lungs looked similar to the healthy mice, with a clear alveolar space and with a low number of micro-metastases that had a small area. In contrast, in the lungs from D2A1-WT-bearing mice, that started to generate a PMN with denser lung structure, more metastases were observed with a larger average area. Thus, conditions where the lung PMN is generated and EMMPRIN levels are increased promote the metastatic cascade, resulting in bigger metastatic lesions. Thus, the EMMPRIN-induced PMN promotes the metastatic cascade.

### 3.9 EMMPRIN induces the STAT3 and ERK1/2 signaling pathways

**Figure 7:**
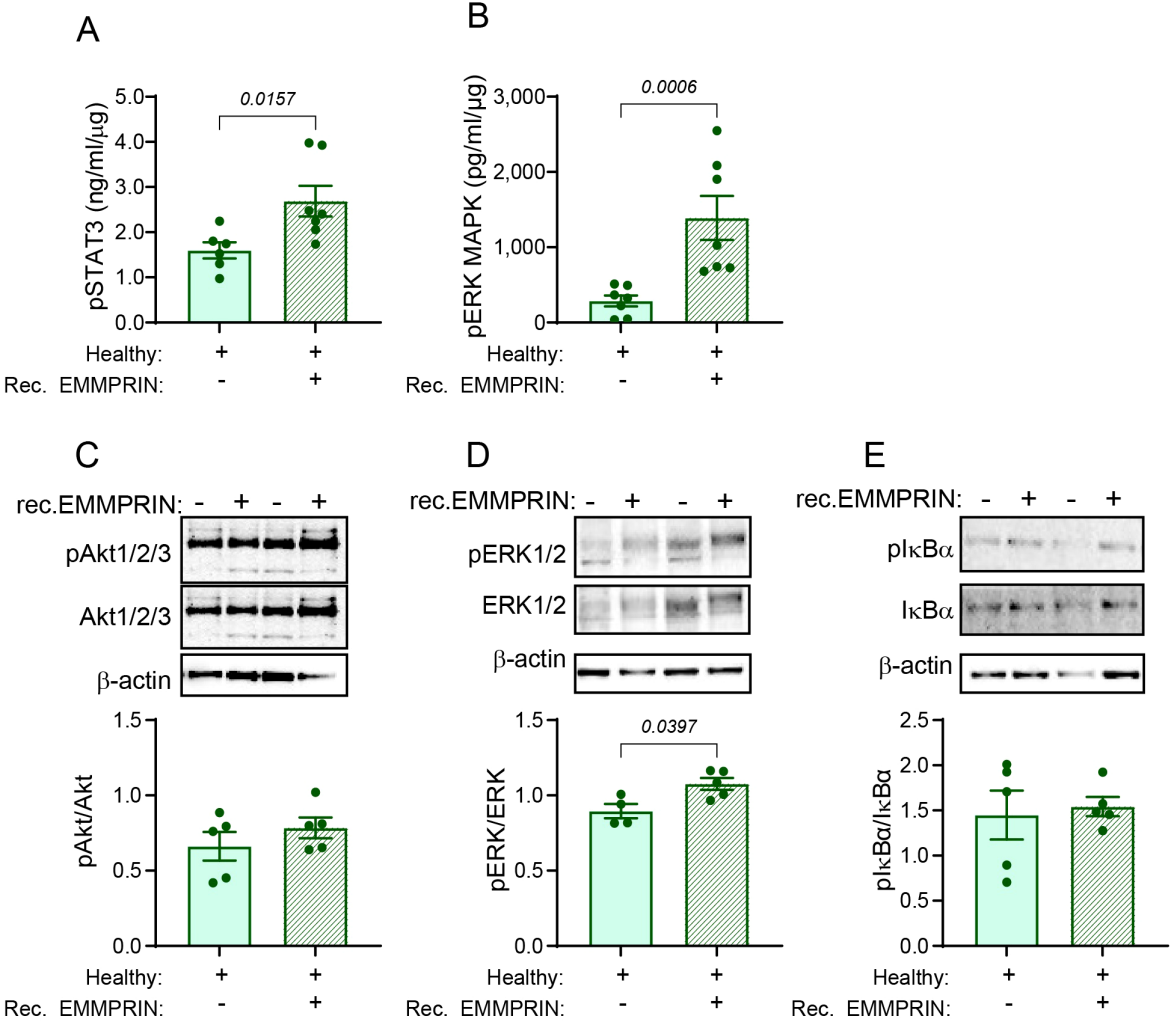
EMMPRIN induces the STAT3 and ERK1/2 signaling pathways. The activation of different signaling pathways was assessed in lung lysates obtained from healthy mice or healthy mice injected with recombinant EMMPRIN from previous experiments, by determining the phosphorylation of key proteins in these pathways. The phosphorylation of **(A)** STAT3 and **(B)** ERK1/2 were evaluated using Intracellular DuoSet ELISA kits (n=7). Additionally, representative images of western blot analyses of two repetitions of the phosphorylation of **(C)** Akt 1/2/3, **(D)** ERK1/2, and **(E)** IκBα, and their quantification (n=5). Recombinant EMMPRIN increased the phosphorylation of STAT3 and ERK1/2, but not of Akt or IκBα, suggesting that the PI3K and the NF-κB pathways do not mediate EMMPRIN’s effect on the lung PMN.

EMMPRIN is known to activate several signaling pathways, including STAT3 (40), PI3K/Akt (24), ERK1/2 MAPKs and NF-κB (24, 25). We, therefore, asked which of these pathways is involved in the generation of the lung PMN. To exclude any possible effects of other factors secreted by the primary tumor, and since we have already shown that administration of recombinant EMMPRIN is sufficient to induce the lung PMN, we examined the activation of these pathways in the lung lysates of healthy mice and healthy mice treated with recombinant EMMPRIN. Analysis of the lysates showed that the STAT3 and the ERK1/2 increase their phosphorylation when recombinant EMMPRIN is administered (**Figures 7A, B**). The activation of the ERK MAPK pathways was further corroborated by a western blot analysis (**Figure 7D**). However, no increase in the phosphorylation of Akt or IκBα was observed (**Figures 7C, E**). Thus, EMMPRIN induces the activation of STAT3 and ERK1/2 to generate the lung PMN.

## 4 Discussion

In this study, we establish that EMMPRIN, as part of the primary tumor secretome, is involved in the generation of the lung PMN, which is a cancer-free but abnormal microenvironment that is different from the primary tumor microenvironment (41), and is a pre-requirement for the later formation of metastases. EMMPRIN has been studied mostly in the context of the primary tumor or in metastasis, and has been found to be a multifunctional protein that participates in many processes that are critical in tumor development (e.g., survival, proliferation, metabolic reprograming, angiogenesis, immune evasion, EMT) (30). However, it has not been investigated before in the context of the PMN. Here, we demonstrate for the first time, that its ability to enhance angiogenesis, activate fibroblasts, recruit immune cells and promote ECM remodeling – all processes that are considered hallmarks of the PMN (12) - contribute to the generation of the lung PMN. Of note, membranal EMMPRIN could also be secreted from the primary tumor packed in extracellular vesicles (EVs) such as exosomes, but here we focused only on the soluble protein.

First, to study the lung PMN we needed to establish an adequate mouse model. The PMN is defined as a normal tissue that does not contain any tumor cells, but is changed in a manner that allows it to become more receptive to future colonizing DTCs (14). Therefore, we needed a model where the primary tumor affects remote organs without DTCs colonizing the site. We chose the D2A1 cell line that was previously described as poorly metastatic, and implanted it orthotopically in immunocompetent mice (33, 37), but we did not resect the primary tumor so as not to enhance spontaneous metastasis. Most mouse models that study the lung PMN sacrifice the mice 2 weeks after implantation (14), but single metastatic cells that colonized the lung early might not be detected as micro- or macro-metastases at 14 days. Therefore, we extended the endpoint to 28 days, to make sure that the lung was free of tumor cells, and that sufficient time was given for the pre-metastatic changes in the lung to become evident. We show by H&E staining and by staining for the D2A1 reporter gene mCherry, that no tumor cells were revealed in the lungs, but the lung itself became dense and exhibited reduced alveolar space, suggesting marked changes in the tissue.

One of the reasons the lung could be free of colonizing DTCs is that the cells lost their metastatic potential. To rule out this possibility, we orthotopically implanted the D2A1-WT cells in immunocompetent mice, resected the primary tumor, and allowed metastases to develop for a long time. Under these conditions, the D2A1-WT cells developed metastases in 50% of the mice, in agreement with previous studies (37), indicating that the cells did not lose their metastatic potential. The strong staining of the primary tumor and the micro- and macro-metastases suggests that we did not encounter a technical problem that could lead to a false negative result. We thus conclude that the absence of tumor cells in the lungs is genuine, validating the new model to study the lung PMN.

We note that the PMN has many characteristics, including enhanced coagulation, complement activation, increased NETosis, and reprogramed glucose and lipid metabolism (11), that we could not examine here due to the enormous scope of such a study. Instead, we chose to focus on some of the central processes and molecules that are necessary for the generation of the lung PMN, such as angiogenesis, neutrophil infiltration, fibroblast activation, and ECM remodeling.

To investigate whether EMMPRIN is involved in the generation of the PMN, we first demonstrated that the primary tumor is the source of elevated serum EMMPRIN levels. We measured the basal serum levels for EMMPRIN in the tumor-free healthy mice. Relative to these basal levels, mice implanted with the D2A1-KD cells show similar levels, whereas mice implanted with D2A1-WT cells have increased levels. This indicates that the primary tumor is the major source of the elevated systemic EMMPRIN levels. Supporting this conclusion is the correlation between serum EMMPRIN levels and tumor weight. These results are in agreement with many studies that have shown increased EMMPRIN levels in the serum of patients with different types of cancer (22, 23, 42–44). It is also possible that EMMPRIN is generated locally in the lung by the resident cells, such as alveolar macrophages, fibroblasts or endothelial cells. However, we did not find changes in EMMPRIN expression in the lung between the different experimental groups (**Supplementary Figure S7**). This may suggest that the increase in EMMPRIN concentrations in the lung lysates obtained from mice implanted with D2A1-WT cells originates from accumulation of serum EMMPRIN and not locally produced by the resident cells, and that the EMMPRIN expressed on the lung resident cell could serve as a receptor for the soluble serum EMMPRIN.

To manipulate EMMRPIN levels, we either knocked-down EMMPRIN expression in the D2A1-KD cells, or neutralized EMMPRIN with the antibody m161-pAb, which we have previously developed in our lab (38). In both cases, EMMPRIN levels in the primary tumor and in the lungs of these mice were reduced. Alternatively, we simulated the increased serum levels of EMMPRIN, by injecting the soluble recombinant EMMPRIN protein, thus matching tumor-derived serum EMMPRIN levels in healthy mice. These three approaches allowed us to manipulate EMMPRIN levels, in order to investigate its contribution to the generation of lung PMN.

We chose to focus on specific pleiotropic cytokines that are central in promoting the PMN. VEGF is a potent pro-angiogenic factor, a chemoattractant to myeloid cells, that enhances the production of inflammatory cytokines, (e.g., TNFα, IL-1β, and IL-6) and promotes immune suppression (45, 46). The pro-angiogenic MMP-9, an ECM-degrading protease, is greatly involved in ECM remodeling and basement membrane degradation, thus allowing endothelial cells to migrate through the ECM to form blood vessels and immune cells to infiltrate the tissue. MMP-9 can also release cytokines and growth factors, particularly VEGF, that are sequestered in the ECM (47, 48). In the lung PMN, TGFβ can enhance angiogenesis and stimulate stroma cells to enhance the deposition of ECM proteins (e.g., fibronectin, Col1), resulting in increased recruitment of immune cells by the fibrotic PMN. TGFβ is a potent immunosuppressive cytokine, as it stimulates T regulatory cell (Tregs), inhibits NK cells, blocks the activation of T cell receptor (TCR), and polarizes tumor-associated macrophages (TAMs) and neutrophils to become M2-like activated macrophages and N2-activated neutrophils (49–51).

EMMPRIN can induce the expression of VEGF and MMP-9 (24, 25), and to be involved in the regulation of TGFβ expression (52). We show that in the lung, EMMPRIN affects the expression levels of these three cytokines, whereas in the primary tumor VEGF and TGFβ are not responsive to changes in EMMPRIN levels. We believe that this discrepancy could result from the effect of additional factors (e.g., hypoxia, acidosis) on the expression of VEGF and TGFβ in the primary tumor that may mask the effects of EMMPRIN. MMP-9, which may be less sensitive to these factors, responds well to EMMPRIN in the primary tumor. Given the chemoattractive properties of VEGF and the ECM remodeling properties of TGFβ and MMP-9, it is likely that EMMPRIN indirectly regulates neutrophil infiltration, fibroblast activation and ECM remodeling by inducing the expression of these three cytokines (and perhaps additional mediators) in lung resident cells. However, it is also possible that EMMPRIN directly enhances neutrophil infiltration and fibroblast activation by binding to its own receptors on these cells. In the current study, we did not delve into the mechanistic details of EMMPRIN’s effect on the lung resident cells, and the determination between these two options merits more investigation in a future study.

While EMMPRIN was clearly involved in the regulation of the lung concentrations of VEGF, MMP-9 and TGFβ, it had no effect on the lung concentrations of the pro-inflammatory TNFα and IL-6 cytokines that have also been implicated in the generation of the lung PMN (2, 53). Although implantation of tumors elevated the lung concentrations of these pro-inflammatory cytokines, neither the D2A1-KD cells, nor the m161-pAb, nor the recombinant EMMPRIN changed their levels. Thus, EMMPRIN selectively regulates cytokines and processes that are important in the generation of the PMN.

The three cytokines regulated by EMMRPIN are necessary to induce angiogenesis and increase vascular permeability, which are pivotal characteristics of the PMN that facilitate colonization of DTCs and immune cell infiltration (2, 15). We demonstrate using the staining for the endothelial cell marker CD31, and the effect of lung lysates on endothelial cell migration during the wound healing *in vitro* assay, that angiogenesis in the lung PMN is indeed enhanced in the presence of high EMMPRIN levels. This is of no surprise, as EMMPRIN is considered a known pro-angiogenic factor in cancerous and inflammatory diseases (54), because of its ability to induce the expression of the potent pro-angiogenic factors VEGF, MMP-9 and TGFβ (24, 25). While it is possible that serum EMMPRIN arrives to the lung and enhances angiogenesis directly by affecting endothelial cells, it can also affect angiogenesis indirectly, by inducing the lung levels of the pro-angiogenic VEGF, TGFβ and MMP-9 secreted by the resident or infiltrating cells, as we have demonstrated, and these two possibilities are not mutually exclusive.

We next chose to examine the infiltration of neutrophils into the lung, as they are pivotal in the generation of the PMN due to their ability to induce inflammation, support tumor cell survival and their extravasation, inhibit immune surveillance, promote angiogenesis and secrete large amounts of MMP-9 (55, 56). Neutrophils can be recruited by chemoattractants, such as CXCL8/IL-8, CXCL12/SDF-1, VEGF, S100A8, and S100A9, secreted both from the primary tumor and from stroma cells in the PMN (13, 55). Here, we demonstrate that EMMPRIN is involved in neutrophil infiltration into the lung PMN, as elevated levels of EMMPRIN, secreted from D2A1-WT tumors or injected as a recombinant protein, promoted neutrophil infiltration, whereas decreased EMMPRIN levels from D2A1-KD tumors or because of neutralization by m161-pAb decreased neutrophil accumulation. Thus, EMMPRIN may be implicated as a new regulator of neutrophil infiltration. This effect could be mediated by the interactions of EMMPRIN with the chemotactic factor cyclophilin A, or via its ability to induce the secretion of the chemoattractants VEGF and SDF-1 (28, 57, 58). In support of the latter mechanism, we have shown that EMMPRIN regulates lung VEGF levels. However, the contribution of cyclophilin A should also be investigated. Importantly, G-MDSC and neutrophils are indistinguishable by genetic differences or by cell surface markers (59). We have used the anti-Ly6G (clone 1A8) antibody, which can stain Ly6G^high^ myeloid cells, including mature neutrophils and G-MSDCs. Therefore, more investigation is warranted to determine whether the stained cells are mature neutrophils or immature G-MSDCs that are more immunosuppressive.

Fibroblasts are necessary in the formation of the PMN. Resident lung fibroblasts can be activated by the tumor secretome or EVs to become cancer-associated fibroblasts (CAFs), that deposit more ECM proteins and ECM regulating enzymes (60). The main cytokine responsible for the fibroblasts activation is TGFβ1 (61), and CAFs can deposit more ECM proteins and secrete cytokines and proteases that recruit immune cells, enhance angiogenesis and remodel the ECM (62–64). Additionally, their crosstalk with endothelial cells enhances the expression of adhesion molecules to facilitate tumor cells colonization (62), and their crosstalk with neutrophils can activate a specific CAF subset that secrets elevated PGE_2_ levels to immunosuppress immune cells (65).

We showed that EMMPRIN regulates TGFβ levels in the lung PMN, suggesting that EMMPRIN could indirectly activate lung fibroblasts via TGFβ. Additionally, EMMPRIN could directly activate fibroblasts via its homophilic interactions. Indeed, enhanced staining of the activation marker αSMA in response to EMMPRIN indicated fibroblast activation. However, because other cell types (e.g., vascular and airway smooth muscle cells, endothelial cells) could also express αSMA, we examined another known fibroblast activation marker, PDGFRα (66), whose mRNA expression was also shown to be regulated by EMMPRIN levels. This is in agreement with a previous study that showed that the activation of quiescent fibroblasts into CAFs by tumor cells is mediated by EMMPRIN, resulting in enhanced EMT and cell migration (67).

In normal lung tissue, resident fibroblasts usually reside beneath the bronchial epithelium and blood vessels (65, 68), and our αSMA staining localizes the fibroblasts in all experimental groups primarily to this peribronchial area, just underneath the epithelial layer. However, since CAFs can originate from many types of cells (e.g., resident fibroblasts, epithelial cells, endothelial cells, pericytes) (69), we cannot determine whether the αSMA-positive fibroblasts originated from resident fibroblast or from epithelial cell undergoing EMT.

ECM remodeling is the net effect of enhanced ECM protein deposition, crosslinking, and degradation. The main cellular compartment responsible for ECM remodeling are fibroblasts that are activated by the primary tumor secretome, and secrete both ECM proteins and regulators, such as LOX or MMPs (15, 70). The lung has a specific ECM composition, where the interstitial matrix that provides the structure and the mechanical strength consists of proteins such as Col1, Col3 and elastin, and their crosslinking by LOX and other enzymes can increase tissue stiffness, cell proliferation and activity of lung fibroblasts (71). The basement membrane, through which DTCs and immune cells infiltrate and that supports their survival and differentiation, consists mostly of Col4, Col6, Col18, fibronectin, and laminins (71). A study that examined the lung PMN in an MDA-MB-231 xenograft model supports the hypothesis that the primary tumor alters ECM proteins in the lung PMN. In contrast to our results, it showed increased expression of collagens (Col4A1 and Col4A5) and degrading enzymes (MMP-2 and MMP-9), whereas LOX levels were reduced (72). The differences in the results could be explained by the absence of T cells in nude mice, which could alter the microenvironmental conditions, or by the difference in tumor sizes (an average of 260 mm^3^ vs. 1,500 mm^3^ in our study), as larger tumors induce hypoxia, which is a strong driver of LOX production (73).

Here, we identify EMMPRIN as a new regulator of ECM remodeling in the lung PMN, with complex effects, as it enhances both the deposition and degradation of ECM proteins in the lung PMN. On the one hand, we show that EMMPRIN regulates the deposition of interstitial collagens, such as Col1A and Col3A, as well as enhances the expression of the crosslinking enzyme LOX, suggesting that EMMRPIN contributes to the increased stiffness of the lung tissue. This is in agreement with the pro-fibrotic activity of EMMPRIN that can stimulate the deposition of Col1 and Col3 (74–77), and that was recently shown to bind to β1 integrins on HT1080 fibroblasts, and stimulate MMPs secretion, TGFβ3 production and deposition of fibronectin and Col5 (78). On the other hand, EMMPRIN enhances the secretion of the ECM degrading enzyme MMP-9, and reduces the protein expression of Col6A, an abundant ECM protein at the lung basement membrane (16, 79). Other basement membrane proteins, such as Col4A, or laminin γ1, are not affected by EMMPRIN, at least at the transcriptional level. The discrepancy between Col6A mRNA and protein expression suggests that the regulatory effect of EMMPRIN is exerted at different levels. While the EMMPRIN-induced signaling cascade promotes the transcription of ECM proteins such as Col1A and Col3A, the enhanced secretion of MMPs, particularly MMP-9, promotes the degradation of the ECM proteins at the protein level. Since MMP-9 is spatially mostly secreted at the basement membrane by infiltrating immune cells, it is reasonable to assume that some basement membrane proteins are degraded more than interstitial collagens, explaining the discrepancy between enhanced overall collagen staining by Masson’s Trichrome and the decreased Col6A protein levels.

To show that EMMPRIN-induced lung PMN indeed supports metastasis, we compared the number and size of metastases generated in an experimental model by i.v. injection of the D2A1-WT cells into healthy mice or mice bearing orthotopic tumors that constantly secrete high levels of EMMPRIN. We show that indeed, mice bearing the orthotopic tumor, which had the chance to develop a lung PMN, had more metastases with a larger area on average, than the group of healthy mice with no lung PMN. Thus, the generation of the lung PMN in our experiments functionally translates into promoting the metastatic cascade, even in early stages.

Lastly, EMMPRIN has been shown to activate many signaling pathways to promote proliferation, angiogenesis, and metastasis (80). Therefore, we asked which EMMPRIN-triggered pathways are involved in the generation of the lung PMN. Comparing between lungs from healthy mice with or without administration of recombinant EMMPRIN, we provide here the first indication that EMMPRIN-induced generation of the lung PMN is mediated through the activation of both STAT3 and ERK1/2, but not via the phosphorylation of Akt or IκBα. This suggests that the STAT3 and ERK MAPK pathways, which have been shown to be co-activated by EMMPRIN (75, 81), are triggered, whereas other pathways, such as PI3K/Akt or NF-κB are not activated in this context. However, our findings are only preliminary, and the identification of the pathways that might be activated by EMMPRIN in the lung with their possible cross-talks, and the identification of the cells affected by EMMPRIN, merits a deeper investigation, which is beyond the scope of this study.

### 4.1 Conclusions

A healthy tissue must undergo significant changes in order to become receptive for colonization by DTCs. While it is accepted that such changes are driven by the remote primary tumor, the components of the tumor secretome responsible for the change have not yet been fully identified and our knowledge of the mechanisms that induce these changes is still incomplete. Here, we provide evidence that EMMPRIN is a part of the tumor secretome. However, it is important to emphasize that this study is only preliminary, identifying EMMPRIN as one of the drivers of the generation of the lung PMN. This opens the door for additional research into the kinetics of the effects of EMMPRIN, its putative effects on additional tissues (which may depend on tissue-specific factors), the molecular signaling pathways that are triggered by EMMPRIN, and the identity of the resident cells that are affected by EMMPRIN and drive ECM remodeling. These questions are interesting and merit additional in depth investigations, which are beyond the scope of this study. Additional confounding factors that might be important and could be controlled by increasing the number of subjects in each experimental group are the tumor size and weight, which varies between mice and depends on the time allowed for the tumor to grow before necropsy, and immune variability that reflects the immune history of each mouse.

We show that the secretion of high levels of EMMPRIN can promote generation of remote lung PMN by simultaneously affecting angiogenesis, immune cell infiltration, and ECM remodeling, all before colonization of DTCs. Thus, we believe that targeting EMMPRIN expression could reduce the ability of DTCs to colonize remote organs. Other antibodies that are already in use or in clinical trials have demonstrated efficacy in targeting EMMPRIN to reduce tumor progression and inhibit metastasis, but the effect on the formation of the PMN was not investigated (31, 32). We demonstrate here that the administration of the new anti-EMMPRIN antibody developed in our lab could inhibit these processes, and specifically hinder the generation of the lung PMN. Thus, targeting EMMPRIN could become a new strategy to prevent the formation of the PMN, and more research is required to establish whether this could in fact reduce metastasis and prevent metastasis-related deaths.

## Data Availability

The original contributions presented in the study are included in the article/**Supplementary Material**. Further inquiries can be directed to the corresponding author.
