# Supplementary material for: Serum EMMPRIN/CD147 promotes the lung pre-metastatic niche in a D2A1 mammary carcinoma mouse model
Source: Front Immunol. 2025 Apr 30;16:1568578. doi: 10.3389/fimmu.2025.1568578 (PMC12075191; doi:10.3389/fimmu.2025.1568578)
Supplement: Supplementary file 1 [file DataSheet1.pdf]

Serum EMMPRIN/CD147 Promotes the Lung Pre-Metastatic Niche in a D2A1 Mammary Carcinoma Mouse Model

Gabriele Feigelman[1], Elina Simanovich[1], Michal A. Rahat[1,2*]

Supplementary Material

Table S1: Four EMMPRIN target sequences used to knockdown its expression

Location*	Gene
364-384	5'- CGACCTGCATACGAAGTACAT
556-576	5'-CCCTCCTATTACAGATTGGTT
611-631	5'- GCAATCACCAATAGCACTGAA
829-849	5'- CCTGGTGTTGGTTACCATCAT

***,** the locations are based on the accession number NP_001070652.1 of mouse EMMPRIN isoform 2

Table S2: list of primers used for qPCR amplification

Gene amplified	Forward primer	Reversed primer
αSMA (ACTA2)	5'- ACCATCGGCAATGAGCGTTTCC	5'- GCTGTTGTAGGTGGTCTCATGG
PDGFRα	5'- GCAGTTGCCTTACGACTCCAGA	5'- GGTTTGAGCATCTTCACAGCCAC
Col1A1	5'- CCTCAGGGTATTGCTGGACAAC	5'- CAGAAGGACCTTGTTTGCCAGG
Col3A1	5'- GACCAAAAGGTGATGCTGGACAG	5'- CAAGACCTCGTGCTCCAGTTAG
Col4A1	5'- ATGGCTTGCCTGGAGAGATAGG	5'- TGGTTGCCCTTTGAGTCCTGGA
Col6A1	5'- GACACCTCTCAGTGTGCTCTGT	5'- GCGATAAGCCTTGGCAGGAAATG
LOX	5'- CATCGGACTTCTTACCAAGCCG	5'- GGCATCAAGCAGGTCATAGTGG
LAMC1	5'- CTGTAATGGGCACAGTGAGACC	5'- ACAAGGCTGGCAGTCAGAGGAG
Basigin (EMMPRIN)	5'- TGGCCTTCACGCTCTTGAG	5'- CAACGCCACTGCTGTTCAAA
GAPDH	5'- CATCACTGCCACCCAGAAGACTG	5'- ATGCCAGTGAGCTTCCCGTTCAG

αSMA, alpha smooth muscle actin; PDGFRα, Platelet-Derived Growth Factor Receptor α; Col, collagen; LOX, lysyl oxidase; LAMC, Laminin subunit γ; GAPDH, Glyceraldehyde 3-phosphate dehydrogenase.

Table S3: List of antibodies used for immunohistochemistry

Protein	Antibody manufacturer	Dilution used
mCherry	Bioss Antibodies, Woburn, MA, USA	1:100
αSMA	Cell Signaling Technology	1:500
CD31	Abcam, Cambridge, UK	1:100
Ly6G (clone 1A8)	Biolegend, San Diego, CA, USA	1:300
HRP-polymer anti-rabbit/rat	Zytomed, Berlin, Germany	20 μL undiluted

Table S4: List of antibodies used for western blot analysis and Immunofluorescence

Protein (application)	Antibody manufacturer	Dilution
Goat anti-EMMPRIN (WB)	R&D systems, Minneapolis, MN, USA	1:1,000
Goat anti-EMMPRIN (IF)	R&D systems, Minneapolis, MN, USA	1:200
Rabbit anti-Collagen 6A1 (WB)	Abclonal, Woburn, MA, USA	1:1,000
Mouse anti-β-actin (WB)	ProteinTech, Rosemont, IL, USA	1:10,000
Donkey anti-goat IgG (WB)	Jackson ImmunoResearch Labs, West Grove, PA, USA	1:5,000
Goat-anti-mouse IgG (WB)	Jackson ImmunoResearch Labs, West Grove, PA, USA	1:5,000
Donkey anti-rabbit IgG (WB)	Jackson ImmunoResearch Labs, West Grove, PA, USA	1:5,000
Donkey anti-goat Alexa Fluor® 568 (IF)	Abcam, Cambridge, UK	1:1,000
ERK1/2 (WB)	Santa Cruz Biotechnology, Dallas, Tx, USA	1:800
Phosphor-ERK1/2 (WB)	Santa Cruz Biotechnology, Dallas, Tx, USA	1:800
Akt 1/2/3 (WB)	Santa Cruz Biotechnology, Dallas, Tx, USA	1:800
Phosphor-Akt 1/2/3 (WB)	Santa Cruz Biotechnology, Dallas, Tx, USA	1:800
IκBα (WB)	Santa Cruz Biotechnology, Dallas, Tx, USA	1:800
Phosphor-IκBα (WB)	Santa Cruz Biotechnology, Dallas, Tx, USA	1:800

This is a provisional file, not the final typeset article

β-actin (WB)	ProteinTech, Rosemont, IL, USA	1:10,000

WB, western blot analysis; IF, immunofluorescence.

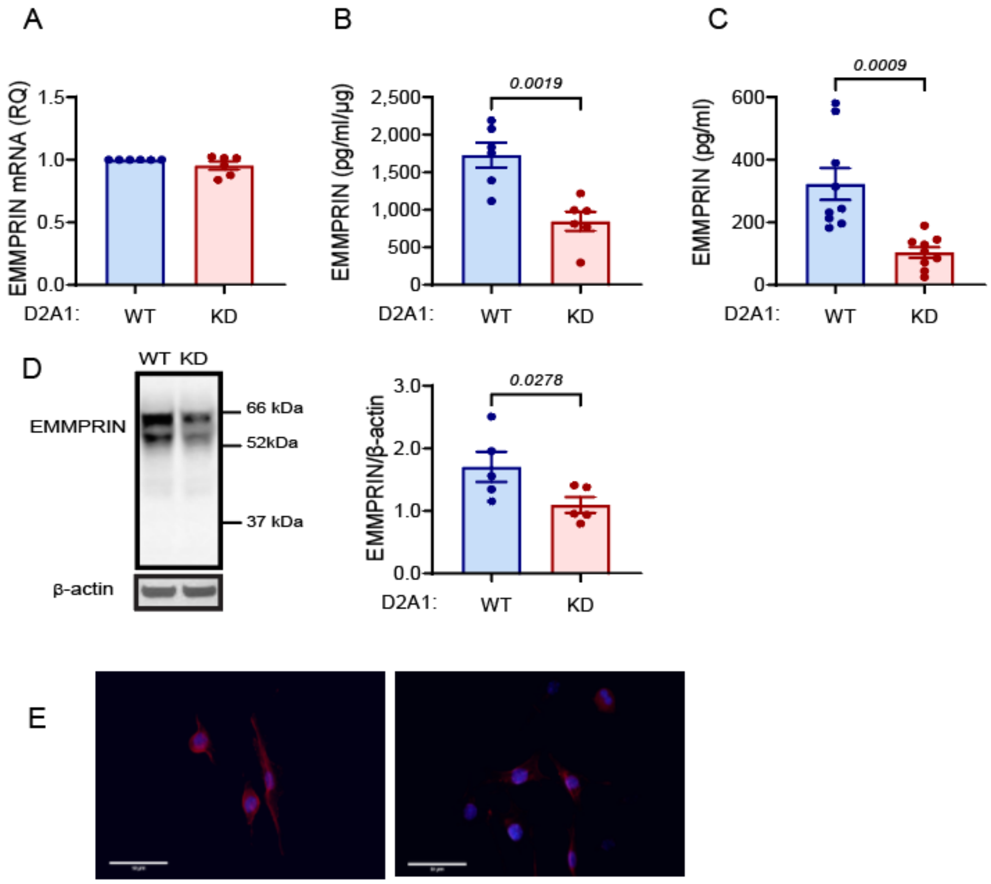

Figure S1. Validation of D2A1-KD cells. The parental D2A1-WT cells and knocked-down D2A1-KD cells were incubated (8×10^4 cells each) in 24-well plates in 400 µL full medium for 48 h. Then (**A**) total RNA was extracted from the cells and amplified using EMMPRIN specific primers (n=6). (**B**) cell lysates (n=6) and **(C)** supernatants (n=9) were collected for an ELISA analysis of EMMPRIN levels. (D) Additionally, cellular levels of EMMPRIN were confirmed by western blot analysis (n=5). The multiple bands reflect the different glycosylation patterns. Data are presented as means ± SE and analyzed using Student's *t* test analysis. (**E**) Representative images of D2A1-WT and D2A1-KD cells (30,000 cells/well/300µL) stained with anti-EMMPRIN antibody as described in the methods, demonstrate reduced EMMPRIN protein expression in the D2A1-KD cells (n=3). Bar size is 20 µM. Although no change is observed in the EMMRPIN mRNA expression, the protein levels in cell lysates or in the supernatants were reduced, suggesting a post-transcriptional regulation.

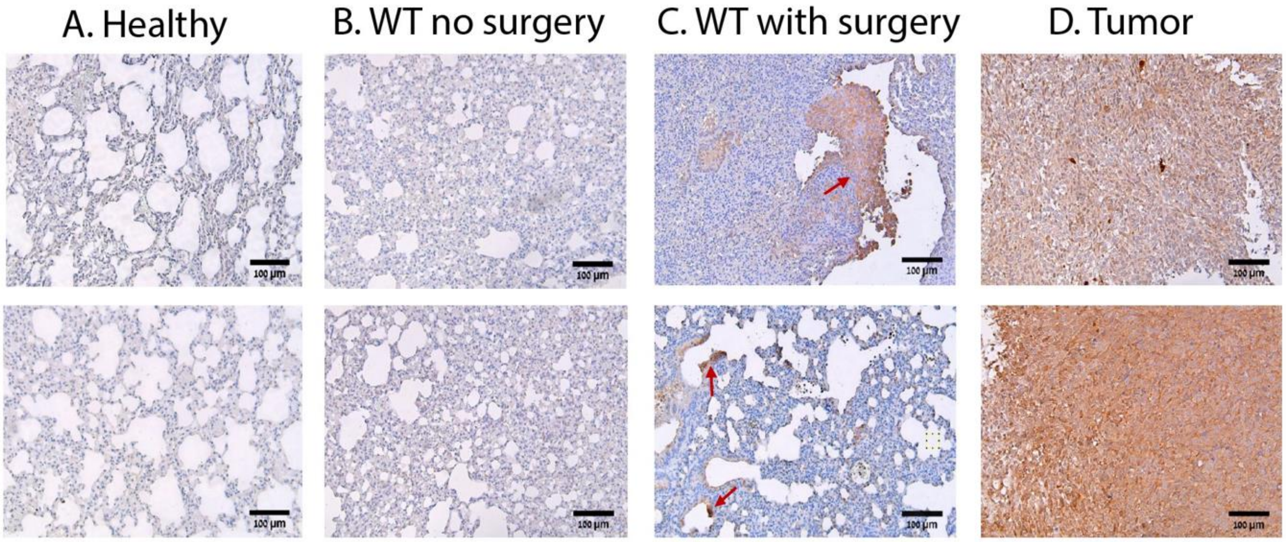

Figure S2. D2A1 cells maintain their metastatic potential. The parental D2A1-WT cells were orthotopically injected (2x10^5) to the fourth mammary fat pad of female BALB/c mice. After 17 days, the implanted tumors were excised (WT with surgery), and mice in this group were sacrificed at day 54 after implantation. Alternatively, the tumors were not excised (WT no surgery) and mice were sacrificed at day 28. Lungs were formalin fixed and paraffin embedded, and lung sections were immunohistochemically stained with the anti-mCherry antibody, as described in the methods. **(A)** Healthy mice; **(B)** Mice implanted with D2A1-WT cells that did not undergo surgery; **(C)** Mice implanted with D2A1-WT cells where the primary tumor was resected; **(D)** Tissue sections from primary tumors. Two representative images from each group are presented. Red arrows point to macro- or micro-metastases. Bar size is 100 μm. Although D2A1-WT cells did not metastasize from the primary tumor to the lung after 28 days, metastases could be identified in the lungs of mice 37 days after the primary tumor was resected, indicating that the cells maintained their potential to metastasize under the right conditions.

This is a provisional file, not the final typeset article

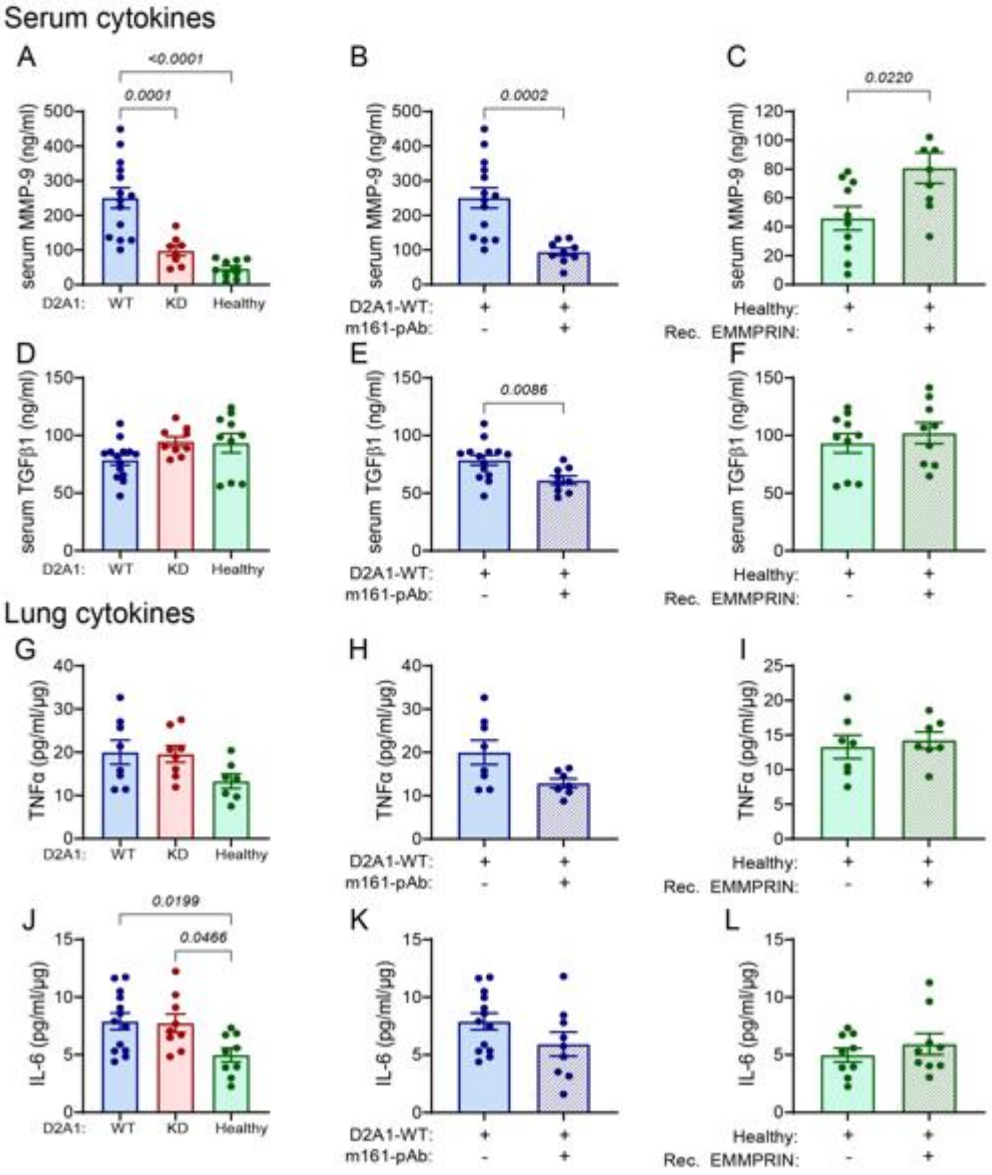

Figure S3: EMMPRIN affects serum MMP-9 and does not affect pro-inflammatory cytokines in the lung. Mice were injected with the different tumor cells and treatments as described in the legend of figure 1. Concentrations of **(A-C)** MMP-9 and **(D-F)** TGFβ in the serum of the different experimental groups were determined using ELISA. Likewise, concentrations of **(G-I)** TNFα and **(J-L)** IL-6 were determined in lung lysates using ELISA (n=13-14 for the D2A1-WT group, n=8-9 for the D2A1-KD group, n=9-10 for the healthy group, n=7,9 for the D2A1-WT + m161-pAb group, n=7,9 for the Healthy + rec. EMMRPIN group). Data are presented as mean ± SEM. Three groups were analyzed using one-way ANOVA followed by Bonferroni's post-hoc test, and two groups were compared using the non-parametric two-tailed Mann-Whitney *t* test. Serum levels of MMP-9 were increased in mice implanted with D2A1-WT cells or injected with recombinant EMMPRIN, whereas serum levels of TGFβ did not change, suggesting that factors other than EMMPRIN may regulate it. The lung levels of TNFα and IL-6 was not affected by the concentrations of EMMPRIN, indicating that these cytokines are not regulated by EMMPRIN.

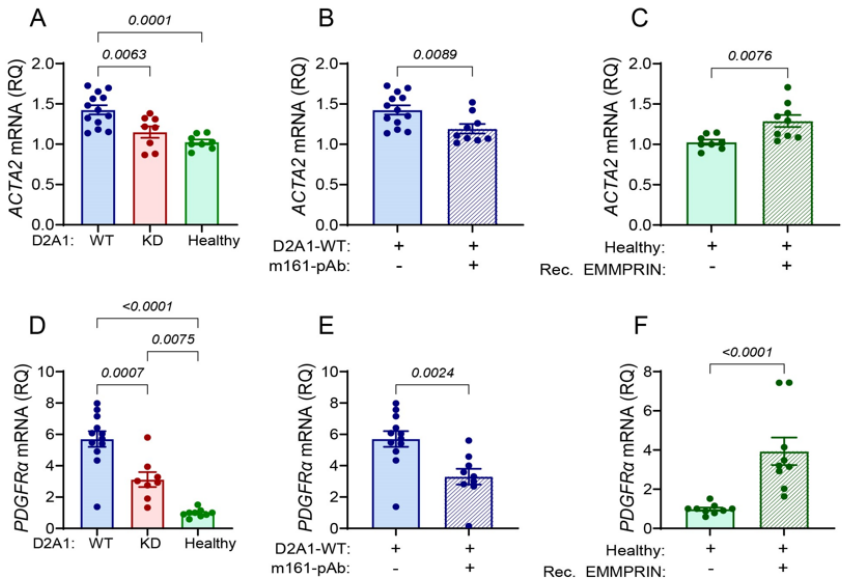

Figure S4: EMMPRIN promotes fibroblast activation. Mice were injected with the different tumor cells and treatments as described in the legend of figure 1. Total RNA was extracted from the lungs, reverse transcribed and amplified for **(A)** the activation marker αSMA (gene name *ACTA2*) and **(B)** the general fibroblast marker *PDGFRα* (n=12-13 for the D2A1-WT group, n=8 for the D2A1-KD group, n=8-9 for the healthy group, n=9 for the D2A1-WT + m161-pAb group, n=8-9 for the Healthy + rec. EMMRPIN group). Data are presented as mean ± SEM. Three groups were analyzed using one-way ANOVA followed by Bonferroni's post-hoc test, and two groups were compared using the non-parametric two-tailed Mann-Whitney *t* test**.** The expression of these two fibroblasts activation markers was increased with high EMMPRIN levels, in mice implanted with the D2A1-WT cells or injected with recombinant EMMPRIN, and reduced in the presence of low EMMPRIN concentrations in mice implanted with the D2A1-KD cells or treated with m161-pAb.

This is a provisional file, not the final typeset article

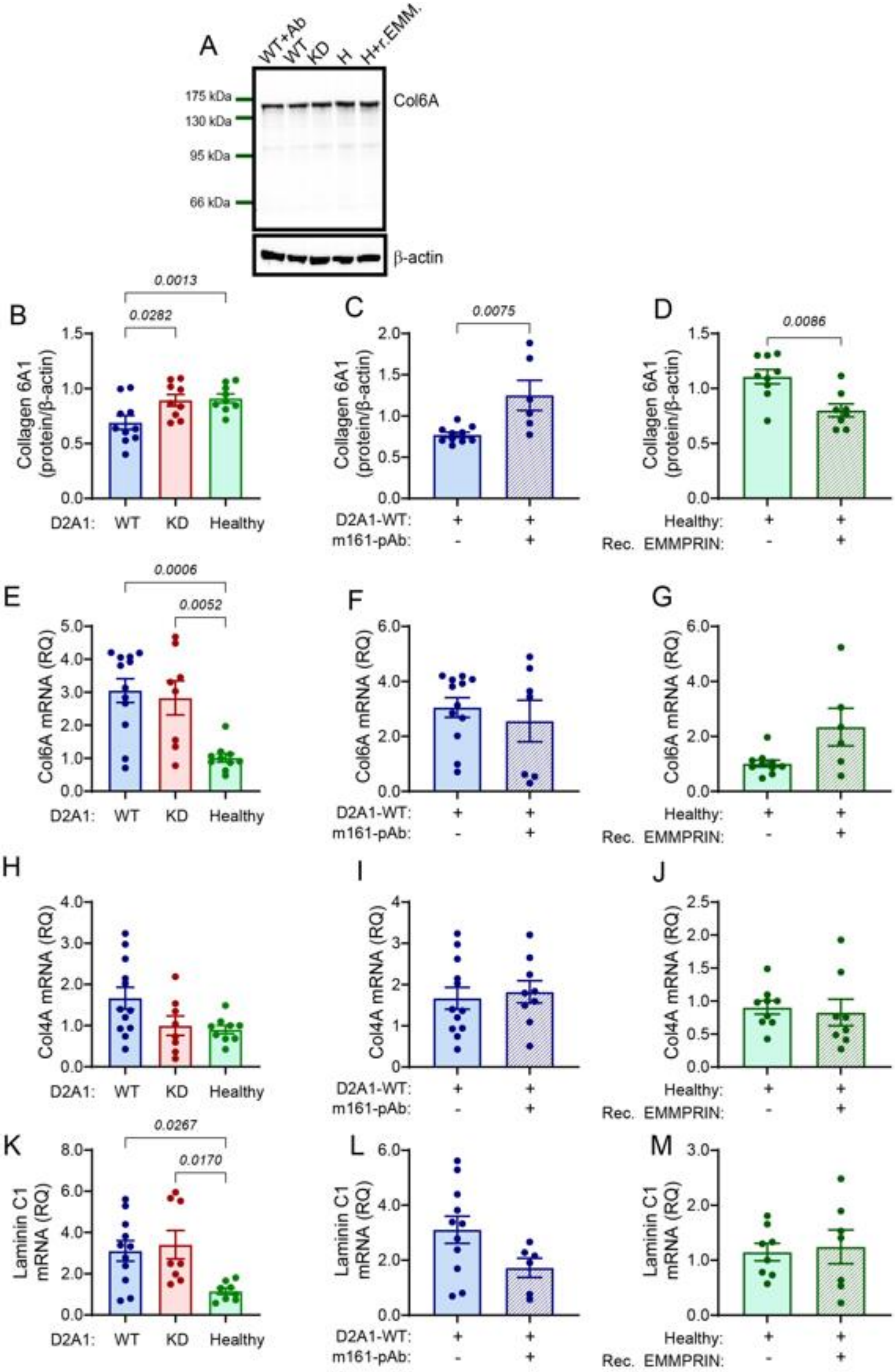
A
WT+Ab
WT
KD
H
H+r.EMM.
175 kDa
130 kDa
95 kDa
66 kDa
Col6A
β-actin
B
0.0013
0.0282
Collagen 6A1 (protein/β-actin)
D2A1: WT KD Healthy
C
0.0075
Collagen 6A1 (protein/β-actin)
D2A1-WT: + +
m161-pAb: - +
D
0.0086
Collagen 6A1 (protein/β-actin)
Healthy: + +
Rec. EMMPRIN: - +
E
0.0006
0.0052
Col6A mRNA (RQ)
D2A1: WT KD Healthy
F
Col6A mRNA (RQ)
D2A1-WT: + +
m161-pAb: - +
G
Col6A mRNA (RQ)
Healthy: + +
Rec. EMMPRIN: - +
H
Col4A mRNA (RQ)
D2A1: WT KD Healthy
I
Col4A mRNA (RQ)
D2A1-WT: + +
m161-pAb: - +
J
Col4A mRNA (RQ)
Healthy: + +
Rec. EMMPRIN: - +
K
0.0267
0.0170
Laminin C1 mRNA (RQ)
D2A1: WT KD Healthy
L
Laminin C1 mRNA (RQ)
D2A1-WT: + +
m161-pAb: - +
M
Laminin C1 mRNA (RQ)
Healthy: + +
Rec. EMMPRIN: - +

Figure S5: EMMPRIN does not directly affect the production of ECM proteins that constitute the basal membrane. Mice were injected with the different tumor cells and treatments as described in the legend of figure 1. **(A)** Lung lysates were collected as described in the methods, and Western blot analysis was carried out for Col6A and **(B-D)** quantified (n=10 for the D2A1-WT group, n=9 for the D2A1-KD group, n=9 for the healthy group, n=6 for the D2A1-WT + m161-pAb group, n=5 for the Healthy + rec. EMMRPIN group). Total RNA was extracted from the lungs, reverse transcribed and amplified for the determination of mRNA expression of **(E-G)** Col6A, **(H-J)** Col4A **(K-M)** laminin γ1/C1 (n=10-12 for the D2A1-WT group, n=8-10 for the D2A1-KD group, n=8-10 for the healthy group, n=6-7 for the D2A1-WT + m161-pAb group, n=5-7 for the Healthy + rec. EMMRPIN group). Data are presented as mean ± SEM. Three groups were analyzed using one-way ANOVA followed by Bonferroni's post-hoc test, and two groups were compared using the non-parametric two-tailed Mann-Whitney *t* test**.** The mRNA expression of proteins that compose the basement membrane was unchanged by the manipulation of EMMPRIN concentrations in the mice implanted with D2A1-KD cells, administered the m161-pAb or injected with recombinant EMMPRIN. In contrast, the protein levels of Col6A were reduced in mice implanted with D2A1-WT cells or injected with recombinant EMMPRIN, and increased when EMMPRIN was neutralized with m161-pAb. Thus, EMMPRIN may post-transcriptionally regulate Col6A.

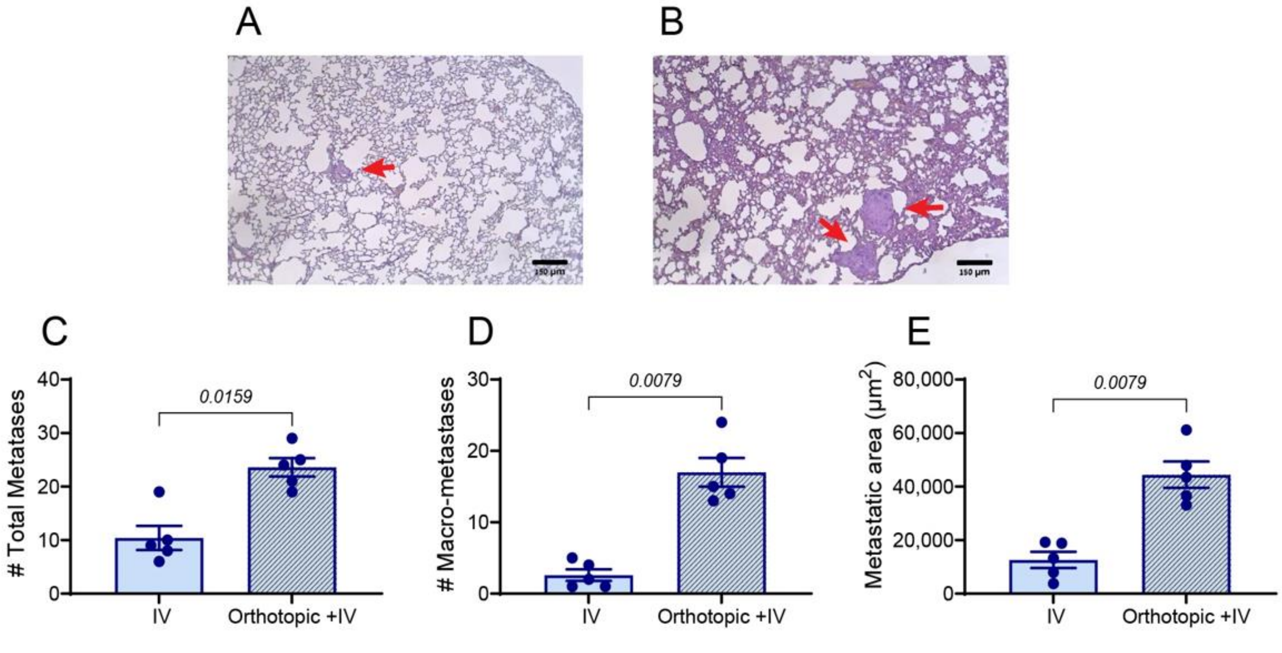

Figure S6: EMMPRIN-induced lung PMN promotes metastasis. Mice were orthotopically injected with D2A1-WT cells (2x10^5 cells) in the 4th mammary fat pad, and tumors were allowed to develop. On day 16, this group or a group of healthy mice were i.v. injected with the D2A1-WT cells (5x10^5 cells), and metastases were allowed to establish until day 25, where both groups were sacrificed (n=5 in each group). Representative images of **(A)** a lung of healthy mouse with i.v. injection of D2A1-WT cells, and **(B)** a lung of D2A1-WT-bearing mouse that was injected i.v. with

This is a provisional file, not the final typeset article

the same cell type. The lungs were formalin fixed and paraffin-embedded, and sections were stained with hematoxylin and eosin (H&E). **(C)** The number of all metastases, **(D)** the number of macro-metastases (defined as >10,000 μm^2), and **(E)** the area of all metastases were measured. Bar size is 150 μm, red arrows point to metastases. The healthy mice show normal alveolar space and have a lower number of metastases with smaller area. In contrast, the D2A1-WT tumor-bearing mice demonstrate denser lung structure, characteristic of the PMN, with macro-metastases that have a larger area on average. Data are presented as mean ± SEM, and the two groups were compared using the non-parametric two-tailed Mann-Whitney *t* test.

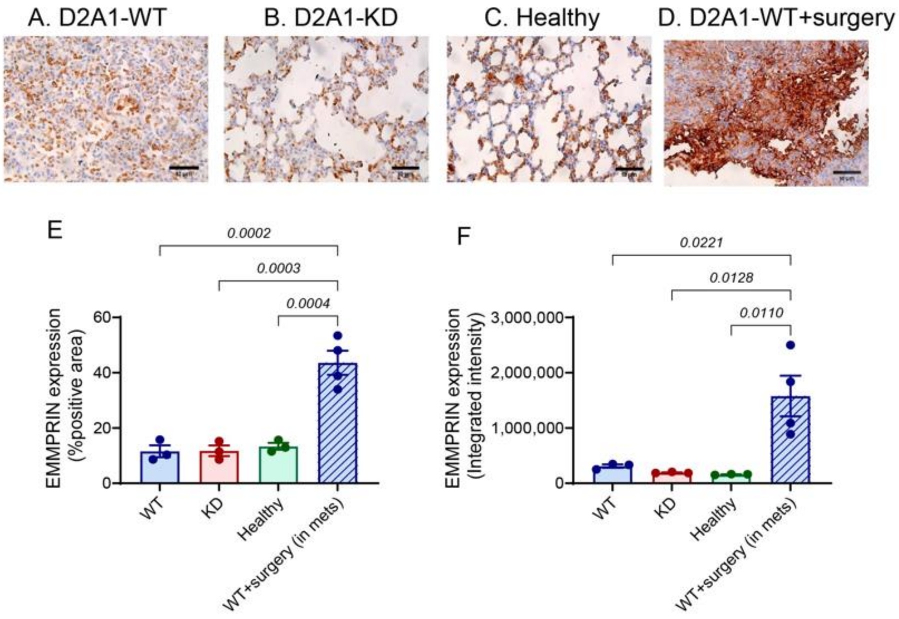

Figure S7: EMMPRIN expression in lung resident cells is unchanged. Representative images of lung tissue sections immunohistochemically stained for EMMPRIN from **(A)** mice implanted with the D2A1-WT cells, **(B)** mice implanted with the D2A1-KD cells, **(C)** healthy mice, and **(D)** mice that were implanted with the D2A1-WT cells and the primary tumor was resected (see legend of figure S2). Images were quantified using he ImageJ software according to (**E**) the percent positive area, and (**F**) the integrated intensity of staining (n=3-4). Data are presented as mean ± SEM, and analyzed using one-way ANOVA followed by Bonferroni's post-hoc test. EMMPRIN is expressed in resident lung cells, but the intensity of the staining remains unchanged in all experimental groups. Only when metastases are generated, an increase in EMMPRIN expression is observed, that is limited to the metastatic cells.
